# Temporal multimorbidity patterns and cluster identification: a longitudinal analysis of administrative data

**DOI:** 10.1186/s12916-025-04184-x

**Published:** 2025-07-01

**Authors:** Jennifer K. Ferris, Brandon Wagar, Alex Choi, Jonathan Simkin, Hind Sbihi, Kari Harder, Kate Smolina

**Affiliations:** 1 BC Centre for Disease Control, Provincial Health Services Authority, Vancouver, BC Canada; 2 Gerontology Research Centre, Simon Fraser University, Vancouver, BC Canada; 3 BC Ministry of Health, Victoria, BC Canada; 4 Vancouver Coastal Health Authority, Vancouver, BC Canada; 5 BC Cancer, Provincial Health Services Authority, Vancouver, BC Canada; 6 School of Population and Public Health, University of British Columbia, Vancouver, BC Canada; 7 Northern Health Authority, Prince George, BC Canada

**Keywords:** Multimorbidity, Comorbidity, Multiple chronic disease, Network, Cluster, Pattern

## Abstract

**Background:**

Multimorbidity is analytically and clinically complex, involving multiple interactions between diseases each with unique implications for health. Identifying disease co-occurrence patterns at the population level could aid in disease prevention, management, and care delivery.

**Methods:**

Here, we analyzed multimorbidity patterns using linked administrative data from a longitudinal cohort of 1,347,820 individuals with multimorbidity over 20 years in British Columbia, Canada. A directed network-based approach was used to assess disease patterns in multimorbidity by frequency (prevalence) and non-random association (lift). We applied a community detection algorithm to identify multimorbidity disease clusters.

**Results:**

Mood and anxiety disorders and hypertension were the most common disease predecessors in prevalence networks, with differences between age groups. Lift networks revealed non-random disease associations. Some indicate potential etiological disease relationships (e.g., breast cancer preceding heart disease in young women), shared risk profiles (e.g., chronic obstructive pulmonary disease and lung cancer), or overlapping disease constructs (e.g., Parkinsonism and dementia). Disease clusters often centered around a single disease as a common predecessor or consequence, representing potential multimorbidity profiles, which may be relevant for patient subgrouping or management.

**Conclusions:**

Insights from these analyses can complement traditional chronic disease surveillance methods, flagging disease patterns for further interrogation into their impacts on function, mortality, and health service utilization.

**Supplementary Information:**

The online version contains supplementary material available at 10.1186/s12916-025-04184-x.

## Background

Multimorbidity, the co-occurrence of two or more chronic conditions, is a critical emerging issue for healthcare systems worldwide [1]. Medical research, disease surveillance, and clinical guidelines have historically operated with a siloed single-disease focus, but co-occurring diseases are highly prevalent and even part of the expected disease course for some chronic conditions [1]. Identifying patterns of disease co-occurrence can provide insights for disease prevention, management, and the coordination of health system services and policies [1–3].

There are two broad categories of disease co-occurrence measures in multimorbidity, each with distinct implications for population health management. The first category measures the most common patterns of disease co-occurrence. Identifying the most prevalent multimorbid disease profiles, and the progression of diseases within them, could inform health care system planning and resource allocation. The second category measures “non-random” disease association, identifying diseases that co-occur more than expected given independent co-occurrence [2, 4]. Non-random co-occurrence may arise from underlying factors that are meaningful in population health monitoring, for example, shared disease etiology, common risk factors, or overlapping disease constructs. The analysis of large-scale disease patterns in multimorbidity can improve chronic disease surveillance by flagging significant disease relationships for further analyses and monitoring.

A network science approach is well-suited for mapping disease patterns in multimorbidity. Networks can visualize complex disease relationships and provide a data backbone to interrogate the structure of disease co-occurrence [5]. Most multimorbidity network studies to date have used undirected networks [4, 6–8], which overlook the temporal sequence of disease occurrence. Directed networks that measure temporality might better model disease progression and interactions [9, 10] and may provide clinically meaningful information on how to interrupt multimorbidity progression. Disease network studies typically consider pairwise disease relationships, but clustering algorithms can additionally be applied to disease networks to identify multi-disease groupings, which better reflects the reality of multimorbidity as a construct. This approach remains underutilized in multimorbidity network research.

The objective of this study was to perform a comprehensive assessment of disease patterns in multimorbidity, with an eye to developing methodologies for application to chronic disease surveillance in health care systems. We employed network analyses and clustering in a longitudinal cohort of individuals with multimorbidity from linked administrative datasets over a 20-year time frame (2001/02 to 2019/20). Diseases were measured with standardized case definitions used for chronic disease surveillance in British Columbia and Canada. We assessed disease patterns in terms of their temporality, frequency (prevalence), and non-random co-occurrence (lift).

## Methods

### Study population and data

This study is a retrospective cohort analysis using linked administrative data from a universal health care system with near-complete population capture. Our study population consisted of residents of British Columbia (BC), Canada (population in 2001/02: 4,076,896 and in 2020/21: 5,226,665) who met the following inclusion criteria: were over the age of 20; were alive and continuously registered in BC for a minimum of 10 years at any point during our study window (fiscal years 2001/02–2020/21); and had multimorbidity, defined as two or more co-occurring chronic conditions. All analyses were stratified by sex and age group (20–44 years, 45–69 years, and 70 + years). Age groupings were determined a priori in consultation with the study team, including clinical input. These age groups provided meaningful age-specific analyses (capturing younger adults, middle aged, and older adults), while also maintaining a reasonable sampling distribution in each age bin. Analyses were conducted using R (V 4.0.5).

We included 25 chronic conditions for multimorbidity analyses: 18 conditions from the BC Chronic Disease Registry and 7 cancer subtypes from the BC Cancer Registry, detailed in Table 1 (see Additional file 1: Supplementary Methods for disease definitions and case counting logic). Since two diseases were sex-specific (breast cancer and prostate cancer), 24 diseases total were included within each sex-stratified group.

**Table 1:** List of diseases in multimorbidity analyses

Data source	Disease	Case definition age cut-off
BC Chronic Disease Registry	Anxiety and mood disorders	1 +
	Asthma	1 +
	Chronic kidney disease	1 +
	Diabetes mellitus	1 +
	Epilepsy	1 +
	Heart failure	1 +
	Osteoarthritis	1 +
	Rheumatoid arthritis	1 +
	Schizophrenia and delusional disorders	10 +
	Gout	20 +
	Hypertension	20 +
	Ischemic heart disease	20 +
	Multiple sclerosis	20 +
	Stroke	20 +
	Chronic obstructive pulmonary disease (COPD)	35 +
	Alzheimer’s and other dementias	40 +
	Parkinson’s disease	40 +
	Osteoporosis	50 +
BC Cancer Registry	Breast cancer	n/a
	Colorectal cancer	n/a
	Hematological cancer	n/a
	Lung cancer	n/a
	Melanoma	n/a
	Other solid organ cancer	n/a
	Prostate cancer	n/a

De-identified linked administrative health datasets used in this study were provided by Population Data BC (PopData BC). Access to data provided by the Data Stewards is subject to approval but can be requested for research projects through the Data Stewards or their designated service providers. The following data sets were used in this study: Chronic Disease Registry, BC Cancer Registry, Consolidation File (Medical Services Plan Registration & Premium Billing), and Vital Events Deaths. You can find further information regarding these data sets by visiting the PopData project webpage at https://my.popdata.bc.ca/project_listings/16–218/collection_approval_dates. All inferences, opinions, and conclusions drawn in this publication are those of the authors and do not reflect the opinions or policies of the Data Stewards.

### Network analyses

Multimorbidity disease patterns were modeled by a disease network, where each disease is represented by a node, and links between nodes are weighted by strength of association between disease pairs (details below). Links are directional, such that a link from node *i* to node *j* indicates an incident case definition for disease *i* was followed by an incident case definition for disease *j*. The BC Ministry of Health Chronic Disease Registry data holdings started operating in 1992/93. To ensure accurate identification of incident cases, our data analyses start after a recommended washout period from 2001/02 onwards [11].

To derive disease network metrics, we first counted all cases of disease co-occurrence for every possible pairing of the 25 included diseases. Disease pair cases were the number of individuals with both disease *i* and disease *j*, considering both temporal orders (*i* then *j* and *j* then *i*). We excluded cases where the incident case definitions occurred on the same day (~ 1.5% of records), as temporality could not be established.

We calculated two metrics for link weight metrics: disease pair prevalence and lift (observed/expected (O/E) ratio). The link weight from disease *i* to disease *j* is denoted as *wij*. Disease pair prevalence was calculated for each directional pair of diseases as a proportion of the total population. Population denominators adhered to the highest age cut-off for each disease pair (see Table 1). Lift (O/E ratio) quantifies the likelihood of observed disease co-occurrence, assuming co-occurrence is random. Directed lift was calculated as follows:

$${\mathrm L\mathrm i\mathrm f\mathrm t\;\left(\mathrm O/\mathrm{ERatio}\right)}_{ij}=\frac{n_{ij}\times N}{n_{ix}\times n_{xj}}$$

where *n*_*ij*_ = the case count of disease *i* followed by disease *j*, *n*_*ix*_ = the case count of disease *i* followed by any other disease, *n*_*xj*_ = the case count of any disease followed by disease *j*, and *N* = the total case counts for all diseases across the entire network [12, 13].

Directional disease pairs were considered a Bernoulli trial, and we retained only links where one direction was statistically significant after Bonferroni correction for multiple comparisons at *p* < 1.70 × 10^−5^ (i.e., where *wij* was significant and *wji* was not significant) [10, 14]. Additionally, we retained only links with lift > 1.0, indicating diseases co-occurred at a higher rate than expected by chance [12, 14]. These filtered pairwise disease links were then used to construct disease networks using the R package *igraph*; networks were visualized with the R package *ggraph*.

We assessed the overall structure of disease networks and the relative importance of diseases within each network. First, we measured *network density*, which is a ratio of the number of actual links in the network compared to the total possible links. Next, we characterized the nodes (diseases) in each network with several metrics. *Node degree* measures the frequency of directional links for each node. *Out degree* is the number of links from each node (i.e., the number of diseases that occur after each disease). *In degree* is the number of links to each node (i.e., the number of other diseases that occur before each disease). *Degree strength* is the sum of the weighted links for each node. *Out strength* is the sum of weighted links from each node, *s*_*i*_^*out*^ = ${\sum }_{x}{w}_{ix}$. *In strength* is the sum of weighted links to each node, *s*_*i*_^*in*^ = ${\sum }_{x}{w}_{xi}$. The maximum node degree corresponds to the number of nodes in the network (maximum node degree = 23). Node strength is the sum of all link weights coming to (in strength) or leaving from (out strength) a given disease node and indexes both the number and prevalence of disease links. Higher node strength indicates more and/or higher prevalence disease links (maximum node strength is 100% prevalence for all 23 links = 2300).

### Disease clustering

Clustering was performed with an algorithm that allows for multiple membership of diseases across clusters and does not force membership of every disease into a cluster. We used the link community detection algorithm implemented in the R package *linkcomm* [15], which clusters the unique links between disease nodes, allowing nodes to belong to multiple communities. Link similarity was indexed by the Tanimoto coefficient [15] and grouped via hierarchical clustering with Ward’s minimum variance. The dendrogram was cut at the height that maximized the Calinski–Harabasz index. Additional details on the link community detection algorithm are provided in Additional file 1: Supplementary Methods. Clusters were named after the disease that appeared most frequently across cluster links.

We calculated disease cluster prevalence by assigning individuals to clusters if they had all the diseases in that cluster in the specified temporal sequence. This is stricter membership criteria than previous multimorbidity studies, which often allow assignment of individuals with only a subset of cluster diseases [16–19]. To balance specificity and stringency, individuals could have additional diseases beyond cluster diseases. Disease cluster prevalence was then calculated as a proportion of the stratified population subgroup, respecting disease-specific age cut-offs. Clusters with fewer than five individuals were excluded to reduce re-identification risk and ensure stable estimates.

## Results

**Fig. 1:**
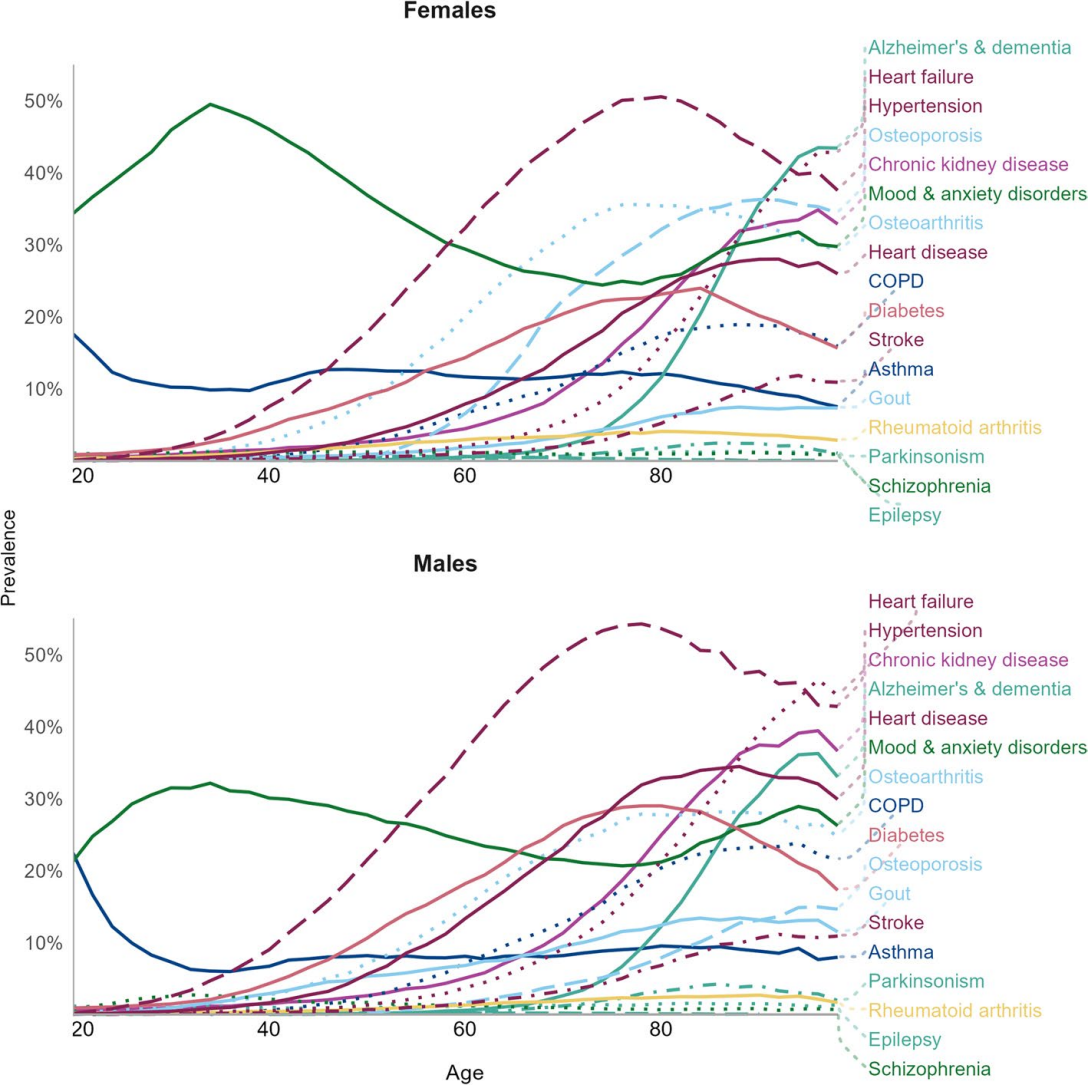
Prevalence of single diseases among general population, by sex and age, in BC, Canada (2001/02–2020/21). Trends in single disease prevalence by sex and age. Linetype and color represent different diseases: diseases are labeled by thin dotted lines, diseases are colored by category (circulatory = wine, diabetes = rose, inflammatory = yellow, kidney disease = purple, mental health = green, musculoskeletal = cyan, neurological = teal, respiratory = blue). Note: Cancers had low prevalence and were excluded from this plot

There were 3,475,352 individuals above the age of 20 with 10 years of continuous registration between 2001/02 and 2020/21 in BC. Of these, approximately 40% had multimorbidity (2 + conditions), leaving a final sample size of 1,347,820. A flow chart of participant inclusion is presented in Additional file 1: Fig. S1. Multimorbidity prevalence increased with age; among individuals aged 20–44, ~ 10% had multimorbidity (females: 12.3%, males: 9.9%), whereas among individuals aged 70 + , ~ 75% had multimorbidity (females: 76.7%, males: 75.1%). Study sample demographics are presented in Table 2. Individual disease prevalence by sex and age are presented in Fig. 1; multimorbidity prevalence by sex and age is presented in Additional file 1: Fig. S2.

**Table 2:** Characteristics of study population

		Study population (*N* = 1,347,820)	
		Females	Males
*N*		702,183	645,637
Age^a^		70 (59–82)	69 (58–79)
Age group^b^	20–44	70,891 (10%)	57,316 (9%)
	45–69	263,899 (38%)	269,937 (42%)
	70 +	367,393 (52%)	318,384 (49%)
Number of diseases^a^		3 (2–4)	3 (2–4)

^a^Median (IQR)

^b^*N* (%)

### Disease prevalence networks

**Fig. 2:**
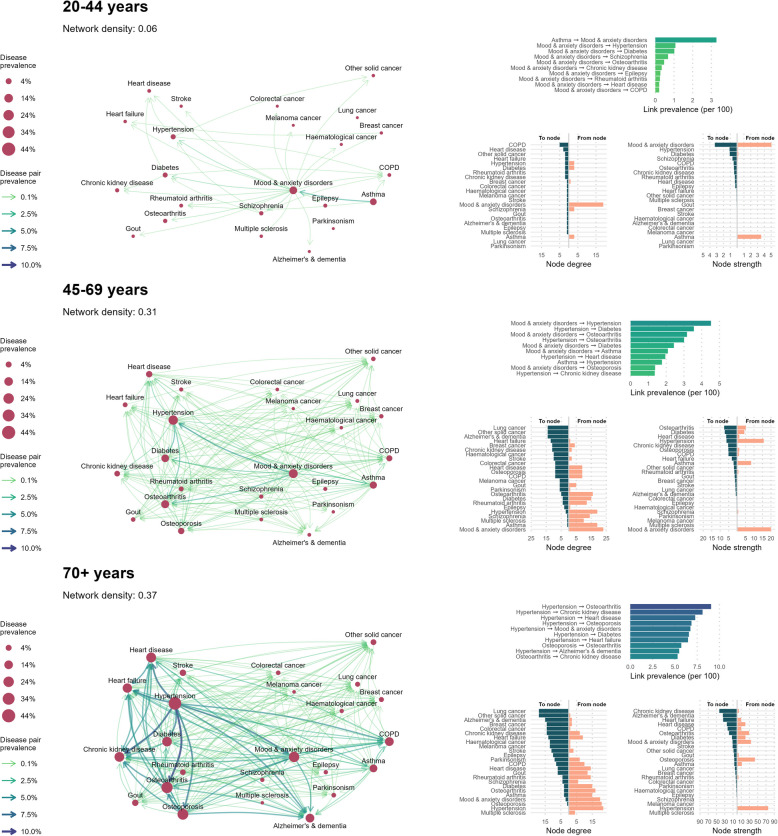
Disease prevalence networks among females with multimorbidity, by age group, in BC, Canada (2001/02–2020/21). Age- and sex-stratified disease prevalence networks for females. Diseases are represented as nodes, and temporal relationships between co-occurring diseases are represented as links. Node size indicates single disease prevalence. Link thickness and color indicates the combined (co-occurring) prevalence of the linked disease pair, and link arrows represent the directionality of disease relationships (first disease–second disease). Network density is a ratio of the number of observed network links over the total possible links. Descriptive information for prevalence networks is presented in the right-hand panel. The top bar plot presents the top 10 most prevalent links in each age-stratified network. The bottom plots present node degree (the number of links coming into or out from each node) and node strength (sum of link weights for links coming into or out from each node)

**Fig. 3:**
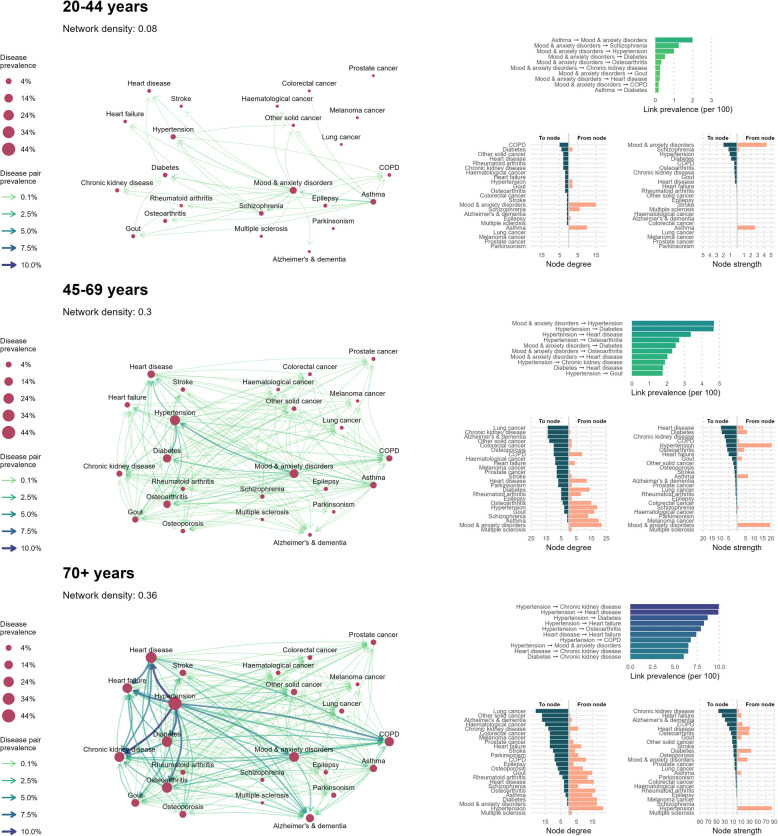
Disease prevalence networks among males with multimorbidity, by age group, in BC, Canada (2001/02–2020/21). Age- and sex-stratified disease prevalence networks for males. Diseases are represented as nodes, and temporal relationships between co-occurring diseases are represented as links. Node size indicates single disease prevalence. Link thickness and color indicates the combined (co-occurring) prevalence of the linked disease pair, and link arrows represent the directionality of disease relationships (first disease–second disease). Network density is a ratio of the number of observed network links over the total possible links. Descriptive information for prevalence networks is presented in the right-hand panel. The top bar plot presents the top 10 most prevalent links in each age-stratified network. The bottom plots present node degree (the number of links coming into or out from each node) and node strength (sum of link weights for links coming into or out from each node)

We characterized common multimorbidity patterns with links weighted by disease pair prevalence. Age-specific disease prevalence networks, along with link and node metrics, are presented in Figs. 2 and 3 for females and males, respectively. The top 15 most prevalent links by sex and age group are provided in Additional file 1: Table S1. Additional file 1: Table S2 contains overall network and node metrics.

#### The most prevalent disease links highlight common multimorbidity patterns

Among individuals aged 20–44, mood and anxiety disorders appeared in nearly all of the top 10 highest prevalence disease links (10/10 for females, 9/10 for males), usually preceding other conditions. The most prevalent link, however, was asthma → mood and anxiety disorders (prevalence among females: 3.27%, among males: 1.99%). Other highly prevalent diseases that occurred after mood and anxiety disorders were schizophrenia (females: 0.68%, males: 1.26%), hypertension (females: 1.07%, males: 1.00%), and diabetes (females: 1.02%, males: 0.52%).

Among individuals aged 45–69, hypertension appeared most frequently in the 10 highest prevalence disease links (6/10 for females and 6/10 for males), followed by mood and anxiety disorders (5/10 for females, 4/10 for males). Hypertension and mood and anxiety disorders usually preceded other conditions. The most prevalent disease link for both sexes was mood and anxiety disorders → hypertension (prevalence among females: 4.52%, among males: 4.69%). Hypertension was commonly followed by diabetes (females: 3.56%, males: 4.66%), heart disease (females: 1.95%, males: 3.36%), and osteoarthritis (females: 3.01%, males: 2.70%). Mood and anxiety disorders were commonly followed by hypertension (females: 4.52%, males: 4.69%), osteoarthritis (females: 3.17%, males: 2.30%), and diabetes (females: 2.43%, males: 2.50%).

Among individuals aged 70 + , hypertension appeared most frequently in the 10 highest prevalence disease links (8/10 of the most prevalent disease links for females, 7/10 for males) and always preceded other conditions. Highly prevalent diseases that followed hypertension were chronic kidney disease (prevalence among females: 8.10%, males: 9.94%), heart disease (females: 7.28%, males: 9.87%), osteoarthritis (females: 9.06%, among males: 7.92%), and diabetes (females: 6.57%, males: 8.68%).

#### Node degree and strength reveal multimorbidity relationships for each disease

Among individuals aged 20–44, chronic obstructive pulmonary disease (COPD) had the highest *in degree* (females: 5, males: 5), while mood and anxiety disorders had the highest *in strength* (females: 3.27, males: 2.00). This means that COPD was preceded by a greater number of different diseases, but mood and anxiety disorders was preceded by more prevalent diseases. Mood and anxiety disorders had the highest *out degree* (females: 19, males: 15) and *out strength* (females: 5.04, males: 4.32). Asthma had high out degree among males, but not among females (females: 3, males: 10), but out strength was higher in females than males (females: 3.54, males: 2.60), indicating that while asthma is followed by a greater number of different comorbid in males, the prevalence of these comorbidities is higher in females.

Among individuals aged 45–69, the nodes with the highest in degree were lung cancer and Alzheimer’s and dementia for both sexes, other solid cancer for females only, and chronic kidney disease for males only (all in degree of 14). The highest in strength node was osteoarthritis for females (7.65) and heart disease for males (9.55). The node with the highest out degree was mood and anxiety disorders (females: 23, males: 22), and the highest out strength node was mood and anxiety disorders for females (20.00) and hypertension for males (20.41). This indicates that mood and anxiety disorders are likely to be followed by many different diseases, but the disorders that follow hypertension among males have slightly higher prevalence than those following mood and anxiety disorders.

Among individuals aged 70 + , the highest in degree node was lung cancer (females: 19, males: 21), tied with other solid cancers for females only (females: 19, males: 17). The highest in strength node was chronic kidney disease (females: 43.22, males: 45.45). This indicates that lung cancer has many different prior disease comorbidities, while chronic kidney disease has fewer but higher prevalence preceding comorbidities. Hypertension was the out degree node (females: 22, males: 22) and highest out strength node (females: 75.31, males: 83.87), emphasizing the significance of hypertension as a predecessor to other comorbid conditions among older adults.

### Lift networks and disease clusters

**Fig. 4:**
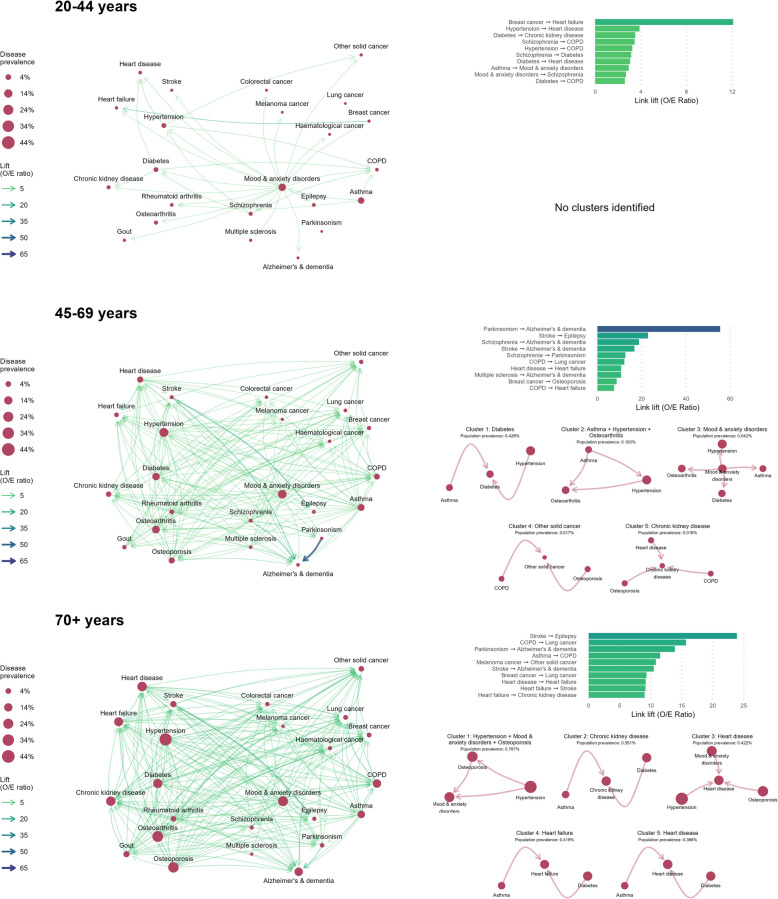
Disease lift (O/E ratio) networks among females with multimorbidity, by age group, in BC, Canada (2001/02–2020/21). Age- and sex-stratified disease lift networks for females. Diseases are represented as nodes, and temporal relationships between co-occurring diseases are represented as links. Node size indicates single disease prevalence. Link thickness and color indicates the lift (observed/expected ratio) of the linked disease pair, and link arrows represent the directionality of disease relationships (first disease–second disease). Descriptive information for lift networks is presented in the right-hand panel. The bar plot presents the top 10 highest lift links in each age-stratified network. The bottom plots present up to 5 of the most prevalent disease clusters identified from each age-stratified lift network. Disease clusters are named after the cluster node with the highest number of links. See Additional file 1: Table S3 for a full list of all disease clusters identified in each network

**Fig. 5:**
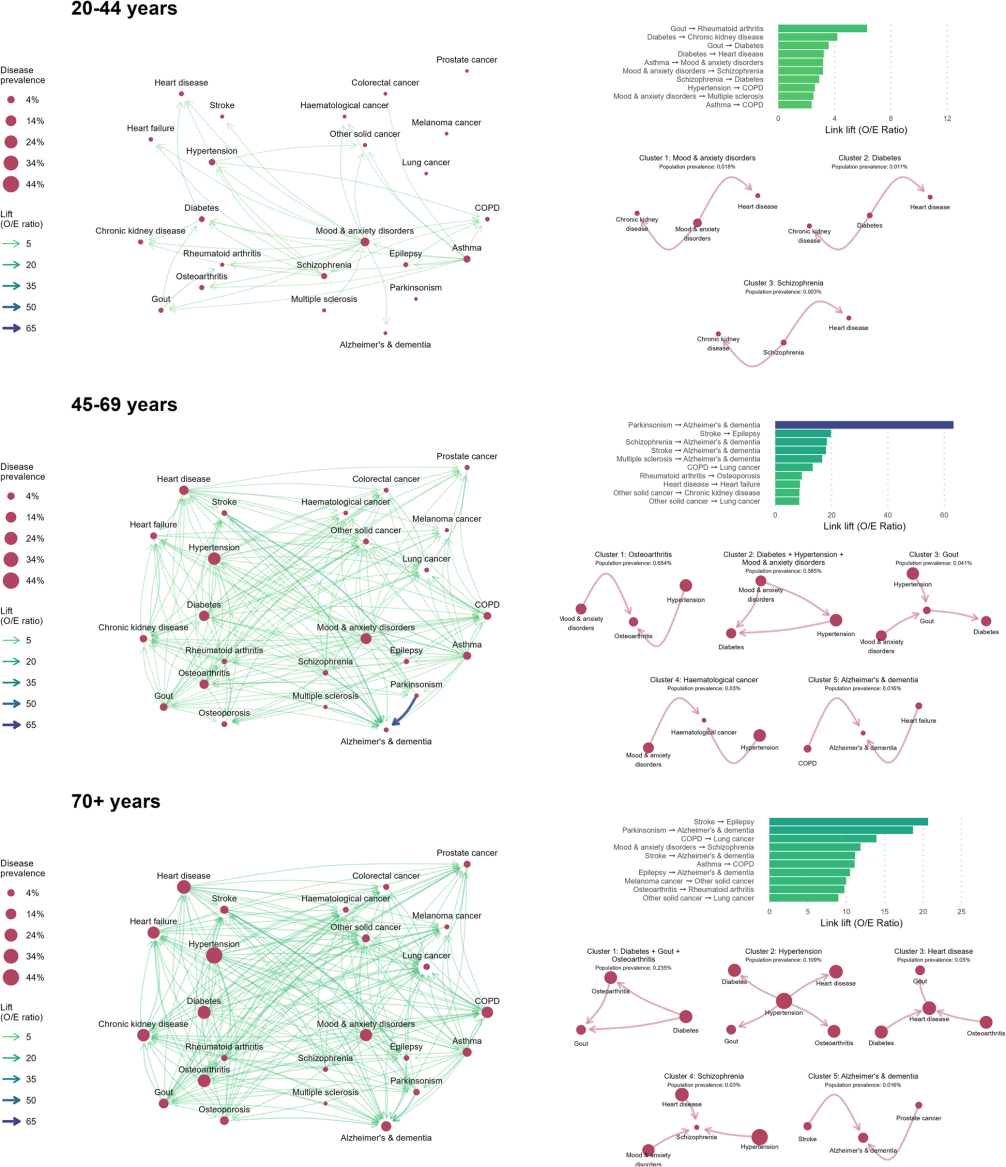
Disease lift (O/E ratio) networks among males with multimorbidity, by age group, in BC, Canada (2001/02–2020/21). Age- and sex-stratified disease lift networks for males. Diseases are represented as nodes, and temporal relationships between co-occurring diseases are represented as links. Node size indicates single disease prevalence. Link thickness and color indicates the lift (observed/expected ratio) of the linked disease pair, and link arrows represent the directionality of disease relationships (first disease–second disease). Descriptive information for lift networks is presented in the right-hand panel. The bar plot presents the top 10 highest lift links in each age-stratified network. The bottom plots present up to 5 of the most prevalent disease clusters identified from each age-stratified lift network. Disease clusters are named after the cluster node with the highest number of links. See Additional file 1: Table S3 for a full list of all disease clusters identified in each network

We characterized non-random disease patterns with links weighted by lift (O/E ratio). Disease clusters were identified from lift networks, and cluster prevalence was calculated. Age-specific lift networks, and the top 5 most prevalent disease clusters, are presented in Figs. 4 and 5 for females and males, respectively. Table 3 provides the total number of disease clusters identified in each sex- and age-stratified network, along with the frequency of each disease across clusters. The 15 highest lift links for each sex and age group are in Additional file 1: Table S3, and detailed information on all identified clusters is in Additional file 1: Table S4.

**Table 3:** Disease frequency in lift network clusters

	Frequency of disease across clusters	Percentage of clusters with disease
20–44 years		
Males		
Number of clusters: 3		
Chronic kidney disease	3	100%
Heart disease	3	100%
Diabetes	1	33%
Mood and anxiety disorders	1	33%
Schizophrenia	1	33%
45–69 years		
Females		
Number of clusters: 27		
Hypertension	15	56%
Asthma	14	52%
Mood and anxiety disorders	13	48%
Osteoarthritis	13	48%
COPD	12	44%
Osteoporosis	12	44%
Diabetes	11	41%
Heart disease	9	33%
Breast cancer	2	7%
Heart failure	2	7%
Lung cancer	2	7%
Multiple sclerosis	2	7%
Other solid cancer	2	7%
Rheumatoid arthritis	2	7%
Schizophrenia	2	7%
Alzheimer’s and dementia	1	4%
Chronic kidney disease	1	4%
Colorectal cancer	1	4%
Epilepsy	1	4%
Gout	1	4%
Hematological cancer	1	4%
Stroke	1	4%
Males		
Number of clusters: 13		
Hypertension	7	54%
Mood and anxiety disorders	7	54%
Chronic kidney disease	4	31%
Diabetes	4	31%
Lung cancer	3	23%
Osteoarthritis	3	23%
COPD	2	15%
Colorectal cancer	2	15%
Gout	2	15%
Heart disease	2	15%
Heart failure	2	15%
Other solid cancer	2	15%
Alzheimer’s and dementia	1	8%
Asthma	1	8%
Hematological cancer	1	8%
Melanoma cancer	1	8%
Rheumatoid arthritis	1	8%
Schizophrenia	1	8%
70 + years		
Females		
Number of clusters: 35		
Hypertension	14	40%
Mood and anxiety disorders	14	40%
Osteoporosis	14	40%
Asthma	12	34%
Diabetes	12	34%
Heart disease	10	29%
Gout	8	23%
Osteoarthritis	8	23%
Chronic kidney disease	6	17%
Heart failure	6	17%
Colorectal cancer	5	14%
Hematological cancer	5	14%
Other solid cancer	5	14%
COPD	4	11%
Lung cancer	4	11%
Rheumatoid arthritis	4	11%
Stroke	4	11%
Alzheimer’s and dementia	3	9%
Breast cancer	1	3%
Schizophrenia	1	3%
Males		
Number of clusters: 11		
Gout	8	73%
Hypertension	7	64%
Osteoarthritis	7	64%
Diabetes	6	55%
Heart disease	5	45%
Alzheimer’s and dementia	2	18%
Asthma	2	18%
Mood and anxiety disorders	2	18%
Osteoporosis	2	18%
Rheumatoid arthritis	2	18%
COPD	1	9%
Chronic kidney disease	1	9%
Heart failure	1	9%
Multiple sclerosis	1	9%
Prostate cancer	1	9%
Schizophrenia	1	9%
Stroke	1	9%

Descriptive information for disease clusters identified from age- and sex-stratified lift networks, including how frequently each disease node appeared across identified network clusters

#### The highest lift disease links highlight non-random disease relationships

Among individuals aged 20–44, the highest lift link for females was breast cancer → heart disease (lift: 12.08), indicating that females with breast cancer were 12 times more likely to develop heart disease than expected based on population prevalence. For males, the highest lift link was gout → rheumatoid arthritis (6.29). Diabetes appeared the most frequently in the top 10 lift links (4/10 for females, 4/10 for males). High lift conditions following diabetes included chronic kidney disease (lift females: 3.51, males: 4.19) and heart disease (females: 3.07, males: 3.22). High lift conditions preceding diabetes were schizophrenia (females: 3.15, males: 2.90) and gout for males (3.58). Other high lift links were hypertension → heart disease for females (3.88) and asthma → mood and anxiety disorders for males (3.18).

Among individuals aged 45–69, the highest lift link was Parkinsonism → Alzheimer’s and dementia (lift females: 55.44, males: 63.28). Alzheimer’s and dementia appeared most frequently across the top 10 lift links (4/10 for females, 4/10 for males), always after a preceding condition. Other high lift diseases that preceded Alzheimer’s and dementia were schizophrenia (females: 18.77, males: 18.34), stroke (females: 16.73, males: 18.02), and multiple sclerosis (females: 10.69, males: 16.66). The second highest lift link was stroke → epilepsy (females: 22.85, males: 19.80).

Among individuals aged 70 + , the highest lift link was stroke → epilepsy (females: 23.88, males: 20.65). For females, stroke and heart failure appeared most frequently across the top 10 lift links (each in 3/10 of the highest lift links). Other notable links involving these conditions were stroke → Alzheimer’s disease (10.49), heart disease → heart failure (9.19), and heart failure → stroke (9.16). For males, Alzheimer’s and dementia appeared most frequently across top lift links (3/10), and high lift diseases preceding Alzheimer’s and dementia were Parkinsonism (18.68), stroke (11.14), and epilepsy (10.48). Another notably high lift link in the 70 + networks was COPD → lung cancer (females: 15.69, males: 13.93).

#### Disease clusters reveal larger groups of non-randomly co-occurring diseases

The number of clusters identified in each age- and sex-stratified disease network ranged from 0 to 35 (see Table 3). Disease clusters typically contained one central disease node (see Figs. 4 and 5 and Additional file 1: Table S3) with other diseases either preceding or following this central node.

Among females aged 20–44, only one cluster was identified, but no individuals met criteria for cluster membership. Therefore, we excluded this cluster result. Among males aged 20–44, three clusters were identified from the lift network and 0.3% of the subgroup was assigned membership to one of these clusters. The most prevalent cluster contained mood and anxiety disorders as a central node, followed by chronic kidney disease and heart disease (cluster prevalence: 0.02% of males aged 20–44).

Among females aged 45–69, 27 clusters were identified and 2% of the subgroup was assigned to a cluster. The most prevalent cluster contained diabetes as a central node, preceded by asthma and hypertension (cluster prevalence: 0.43%). Among males aged 45–69, 13 clusters were identified and 4% of the subgroup was assigned to a cluster. The most prevalent cluster contained osteoarthritis as the central node, preceded by hypertension and mood and anxiety disorders (cluster prevalence: 0.65%).

Among females aged 70 + , 35 clusters were identified and 4% of the subgroup was assigned to a cluster. The most prevalent cluster lacked a central node, with hypertension, osteoporosis, and mood and anxiety disorders appearing with equal frequency (cluster prevalence: 0.77%). Among males aged 70 + , 11 clusters were identified and 0.6% of the group was assigned to a cluster. The most prevalent cluster contained diabetes, osteoarthritis, and gout, appearing with equal frequency (cluster prevalence: 0.24%).

We examined the frequency of disease appearance across clusters (see Table 3). Among males aged 20–44, chronic kidney disease and heart disease were present in all three disease clusters. Among individuals aged 45–69, hypertension appeared in more than 50% of disease clusters (females: 56% of clusters, males: 54%). Asthma appeared in 52% of female clusters and mood and anxiety disorders in 54% of male clusters. Among females aged 70 + , hypertension, mood and anxiety disorders, and osteoporosis each appeared in 40% of identified clusters. For males aged 70 + , gout was the most frequently appearing disease across clusters (73% of clusters), followed by hypertension and osteoarthritis (each in 64% of clusters).

## Discussion

By leveraging network analysis and disease clusters, we provide novel insights into the population dynamics of multimorbidity. Our results highlight the variability and complexity of multimorbid disease patterns and emphasize the benefits of incorporating multiple measures of disease association. The strengths of our study include the use of large administrative datasets with standardized case definitions, multiple measures of multimorbidity relationships, and a well-grounded approach to disease clustering methodology. This approach can serve as a blueprint for developing enhanced chronic disease surveillance strategies.

### The most prevalent patterns of multimorbid disease co-occurrence

Using the prevalence of disease co-occurrence as a network link weight revealed typical multimorbidity disease progression in the population. Because these patterns are a function of single disease prevalence, they are often unsurprising, but there remains value in quantifying typical multimorbid disease profiles. This information can aid health systems planning, for instance in improving projections of health system burden in aging populations.

Highly prevalent multimorbid diseases mirrored the observed age-dependent prevalence trends of single diseases, with mood and anxiety disorders dominating prevalence networks in younger adults and hypertension dominating older adult networks. Both conditions were often predecessors to other multimorbid diseases, particularly in older adult networks (ages 45–69 and 70 +). Hypertension also had high disease network connectivity in previous non-temporal multimorbidity disease network research [20, 21]. Our findings emphasize the importance of hypertension and mood and anxiety disorders as “entry points” into multimorbidity, which may be explained by typical life course profiles (mood and anxiety disorders are highly prevalent in younger populations), increased risk of subsequent chronic conditions [22, 23], and/or ease of screening and diagnosis for these conditions (leading to high detection rates and potential increased monitoring for other comorbid conditions).

Other diseases were more likely to occur after a previous condition; these included lung cancer, chronic kidney disease, heart disease, COPD, osteoarthritis, and Alzheimer’s disease and dementia. These diseases have older ages of onset, and in some cases high mortality, meaning it is more likely that comorbid conditions will precede the incident case. However, in some cases preceding conditions are direct risk factors for the disease of interest, such as diabetes being a significant risk factor for chronic kidney disease [24], heart disease [25], and Alzheimer’s disease and dementia [26]. Knowledge of these disease patterns could improve risk assessments for later-life diseases (such as dementia) and improve clinical management by anticipating coordination between specialists.

### Non-random multimorbidity patterns and disease clusters

Using lift as a measure of disease co-occurrence revealed distinct multimorbidity patterns compared to those identified through disease prevalence networks. High lift conditions likely co-occur for meaningful reasons, regardless of individual disease prevalence. The disease clusters identified from lift networks typically featured a central disease node as a predecessor or follower of other diseases, indicating that clusters are capturing patterns of multimorbidity progression. Although disease clusters had low overall prevalence due to our stringent membership criteria, they represent specific multimorbidity subgroups that highlight important disease relationships for further investigation.

We can hypothesize about the underlying contributors to lift network findings. Some patterns may reflect shared etiological pathways. For instance, cluster #5 in males aged 70 + included stroke and prostate cancer preceding Alzheimer’s disease and dementia. Stroke is known to directly cause dementia [27], and androgen deprivation therapy for prostate cancer has also been associated with an elevated risk of dementia [28]. This cluster may represent a patient profile at higher risk of dementia due to compounding risk from these comorbidities. Another potential etiological pattern was observed in females aged 20–45, with a high lift value from breast cancer → heart disease. Given that breast cancer therapies can damage heart tissue [29], and considering the relatively young onset of heart disease in this age group, this network link likely indicates an etiological disease relationship.

Some patterns may reflect common risk factors. For example, the high lift link between COPD → lung cancer in older adults (40–69 and 70 + years) might be attributable to the shared behavioral risk of smoking [30]. Smoking could also explain cluster #14 identified among females aged 40–69, which included COPD along with heart disease [31] and osteoporosis [32] as predecessors to lung cancer. Another example is cluster #2 among males aged 45–69, with mood and anxiety disorders preceding hypertension and diabetes. This is consistent with behavioral [33] or pro-inflammatory [34] risk factors connecting mental health disorders and cardiometabolic conditions. Finally, cardiovascular and metabolic diseases frequently had high lift values and clustered together (e.g., cluster #2 among males aged 20–44 years old, with diabetes preceding chronic kidney disease and heart disease). This is in line with well-established shared etiological and risk factors between cardiovascular and metabolic diseases, and these patterns have been observed in previous multimorbidity disease clustering research [20, 21, 35].

Some patterns might be explained by an overlap in disease constructs. Notably, the link between Parkinsonism → Alzheimer’s and dementia in adults aged 45–69 was the highest lift value observed across all networks. This relationship has also been reported in previous Canadian administrative data research [36], where elevated dementia risk persisted for several years after incident Parkinsonism. However, the case algorithm for Alzheimer’s and dementia includes International Classification of Diseases, Tenth Revision (ICD-10) code F02.3: Dementia in Parkinson’s disease. Therefore, it is likely that this disease relationship reflects individuals developing dementia as part of the expected disease progression of Parkinson’s. This represents overlapping disease constructs rather than a classic multimorbidity relationship between distinct disease entities. It is important to carefully consider how disease entities are defined in multimorbidity research, because this has a significant impact on observed results.

We have highlighted the above disease relationships because they represent known disease patterns and thus substantiate the validity of our cluster analyses. However, our results also flagged additional disease relationships that are less well-established and may represent new avenues for exploration. These include asthma appearing across multiple clusters as a predecessor to various cardiometabolic conditions among females (diabetes, hypertension, heart disease, and heart failure), schizophrenia as a predecessor to Parkinsonism at 13 times the expected rate among females aged 45–69, epilepsy as a predecessor to Alzheimer’s disease and dementia at 10 times the expected rate among males aged 80 + , and the frequent appearance of gout and osteoarthritis in disease clusters among men aged 80 + . These results demonstrate how network and cluster analyses can flag potentially meaningful multimorbidity relationships for further interrogation.

It is difficult to compare our results with the few existing multimorbidity network clustering studies, primarily because of the different granularity of disease definitions applied. Our study uses defined disease entities with validated case algorithms that are employed in routine national and provincial chronic disease surveillance. In contrast, most previous network clustering studies have used highly granular 3- or 4-digit level ICD codes (or similar) as the unit of analysis [6, 10, 13, 37, 38]—a level of precision that routine administrative data often cannot support. A result of this high granularity is that disease clusters are often composed of related disease codes, due to their overlap in disease constructs (i.e., disease clusters contain codes from the same ICD chapter or larger disease entity), whereas our results evaluate relationships between distinct disease entities. Additionally, previous studies are highly heterogeneous in their choice of link weights, clustering algorithm, and consideration of disease temporality. Consensus and standardization in methodologies are needed to enable comparable analyses and establish the replicability of multimorbidity disease patterns [39, 40].

### Limitations

Administrative datasets are ideal for multimorbidity analyses due to their large size, extensive longitudinal timeframes, standardized disease definitions, and non-biased sampling frames, reflecting comprehensive population coverage. Our analysis comprised of a well-established and standardized list of chronic diseases used in routine chronic disease surveillance. The case algorithms for disease definitions are nationally and provincially standardized and show moderate to high validity [41, 42]. However, case definitions rely on access to and use of health care services; thus, detection may be impacted by broader diagnostic/health care access bias. Furthermore, the incident case date may not correspond the true disease onset, and underdetection of disease is possible. Our study methodology could be employed in future research to interrogate relationships with additional chronic diseases that were not available in the current analysis. Importantly, our analyses are generalizable only to similar disease lists with similar granularity in definitions. A different set of diseases would necessarily yield different findings in the resulting disease networks. We required 10 years of continuous registration in BC public health care system for study inclusion; this strengthened our ability to detect temporal disease relationships; however, it may limit inclusion of populations with higher mobility, such as younger adults or immigrants. Our analysis was stratified by binned age groups; while our age bins were carefully selected a priori with clinical input, our results are also specific to these age bins and findings may change with different age strata. We only included disease relationships data in a single temporal direction, meaning we did not capture the total (undirected) population burden of multimorbidity. We did not include indexes of social marginalization, including race and ethnicity. Previous studies have shown distinct multimorbidity profiles between different racialized groups [4, 7]. Social marginalization significantly impacts health care access and delivery, which in turn will affect pattern detection in multimorbidity. This is a critical avenue for future research.

## Conclusions

This study offers a comprehensive assessment of temporal multimorbidity disease patterns, in disease prevalence, non-random disease co-occurrence, and disease clustering. We identified several significant disease relationships in multimorbidity, which warrant further exploration to understand their impacts on social and physical function, mortality, and health service utilization. Identifying multimorbid disease patterns will improve assessment of multimorbidity burden and inform its management.

## Supplementary Information

Additional file 1: Supplementary materials accompanying this manuscript. Contains Supplementary Methods, Figures S1 and S2, and Tables S1–S4. Fig. S1 Participant inclusion flow chart. Fig. S2 Prevalence of co-occurring diseases among general population, by sex and age, in BC, Canada (2001/02–2020/21). Table S1 Directed link data for top 15 most prevalent network links. Table S2 Network descriptive statistics. Table S3 Directed link data for top 15 highest lift network links. Table S4 Lift network clusters.

## Data Availability

De-identified linked administrative health datasets used in this study were provided by Population Data BC (PopData BC).  Access to data provided by the Data Stewards is subject to approval but can be requested for research projects through the Data Stewards or their designated service providers. The following data sets were used in this study: Chronic Disease Registry, BC Cancer Registry, Consolidation File (Medical Services Plan Registration & Premium Billing), and Vital Events Deaths. You can find further information regarding these data sets by visiting the PopData project webpage at: https://my.popdata.bc.ca/project_listings/16-218/collection_approval_dates. All inferences, opinions, and conclusions drawn in this publication are those of the authors, and do not reflect the opinions or policies of the Data Stewards. The datasets generated and/or analyzed in the current study are not publicly available to protect against any re-identification risk but are available from the corresponding author on reasonable request.

## Abbreviations

BC: British Columbia
COPD: Chronic obstructive pulmonary disease
ICD: International Classification of Diseases
