# Supplementary material for: Temporal multimorbidity patterns and cluster identification: a longitudinal analysis of administrative data
Source: BMC Med. 2025 Jul 1;23:370. doi: 10.1186/s12916-025-04184-x (PMC12219922; doi:10.1186/s12916-025-04184-x)
Supplement: Supplementary file 1 — Additional file 1: Supplementary materials accompanying this manuscript. Contains Supplementary Methods, Figures S1 and S2, and Tables S1–S4. Fig. S1 Participant inclusion flow chart. Fig. S2 Prevalence of co-occurring diseases among general population, by sex and age, in BC, Canada (2001/02–2020/21). Table S1 Directed link data for top 15 most prevalent network links. Table S2 Network descriptive statistics. Table S3 Directed link data for top 15 highest lift network links. Table S4 Lift network clusters. [file 12916_2025_4184_MOESM1_ESM.docx]

Temporal multimorbidity patterns and cluster identification: A longitudinal analysis of administrative data

Supplementary Methods

## Disease definitions and case counting

### BC Chronic Disease Registry diseases

The BC Chronic Disease Registry (BCCDR) uses the following administrative health databases to identify chronic conditions: Discharge Abstract Database, Medical Services Plan, and PharmaNet. Chronic diseases are identified based on validated case algorithms adapted from the Canadian Chronic Disease Surveillance System [1].

We selected 18 diseases for multimorbidity analyses from 25 available conditions in the BCCDR (as of Version V2020). For BCCDR diseases, prevalent cases were defined as lifetime prevalence from the incident case definition.

### BC Cancer Registry diseases

The BC Cancer Registry is a dataset of all new reportable cancers diagnosed among BC residents. Data sources for cancer records include, but are not limited to, pathology laboratories, vital statistics, abstract summaries of patient hospital stays, health records from cancer treatment centres, among others. We included all invasive cancers, as well as in-site bladder, following the inclusion criteria of the Canadian Cancer Statistics publication[2]. We created seven cancer groupings for multimorbidity analyses, reflecting the most common cancers (breast, prostate, lung, colorectal) and those with similar etiology or disease characteristics (melanoma, blood, and all other solid organ cancers, see Table 1) consistent with the Canadian Cancer Statistics groupings [2].

We defined prevalent cancer cases as 5-year period prevalence from the incident case definition to account for possible cancer remission. When determining cases by cancer type, tumours of the same type that did not overlap in 5-year case windows were counted as separate cases. When tumours overlapped in 5-year case windows, we extended the case window from the first incident tumour case to the end of the 5-year window for the most recent tumour case. We counted only the first occurrence of a cancer of the same type as the incident case for multimorbidity incidence case counts.

## Link Community Detection algorithm

To detect disease clusters from our network, we applied the link community detection algorithm developed by Ahn *et al* (2010)[3] implemented in the R package *linkcomm*[4]. The link community detection algorithm clusters the unique links between nodes based on the proportion of neighbours shared between links.[3] In other words, the *relationships* between diseases are clustered, rather than the diseases themselves. This allows for temporally specific disease relationships, and for diseases to appear across multiple clusters (rather than being forced membership in a single cluster only). The Ahn algorithm is applied to weighted networks using an extension of the Jaccard index: the Tanimoto coefficient.[4] Weighted link similarity between link *e_ik_* and link *e_jk_* is defined as:

$$S\left( e_{ik}, e_{jk} \right)= \frac{a_{i}\bullet a_{j}}{\left| \left. a_{i} \right| \right.^{2}+\left| \left. a_{j} \right| \right.^{2}- a_{i}\bullet a_{j}}$$

where *a_i_* is a vector of the link weights between node *i* and all other nodes directly connected to both nodes *i* and *j*. [4] Link similarity is the dot product of *a_i_* and *a_j_* divided by the squared norms of *a_i_* and *a_j_* minus the dot product.[4] Link similarity is then clustered with a hierarchical clustering algorithm with Ward’s minimum variance; each link forms a leaf in the dendrogram and branches are link communities.[3] To obtain disease clusters, we cut the dendrogram at the height maximizing the Calinski–Harabasz index.

# References

1. Lix L, Ayles J, Bartholomew S, Cooke C, Ellison J, Emond V, et al. The Canadian Chronic Disease Surveillance System: A model for collaborative surveillance. Int J Popul Data Sci. 2018;3:1–11.

2. Canadian Cancer Statistics Advisory Committee in collaboration with the Canadian Cancer Society Statistics Canada and the Public Health Agency of Canada. Canadian Cancer Statistics 2023. 2023. http://cancer.ca/Canadian-Cancer-Statistics-2023-EN. Accessed 5 Jun 2024.

3. Ahn YY, Bagrow JP, Lehmann S. Link communities reveal multiscale complexity in networks. Nature. 2010;466:761–4.

4. Kalinka AT, Tomancak P. linkcomm: An R package for the generation, visualization, and analysis of link communities in networks of arbitrary size and type. Bioinformatics. 2011;27:2011–2.

# Supplementary Figures and Tables


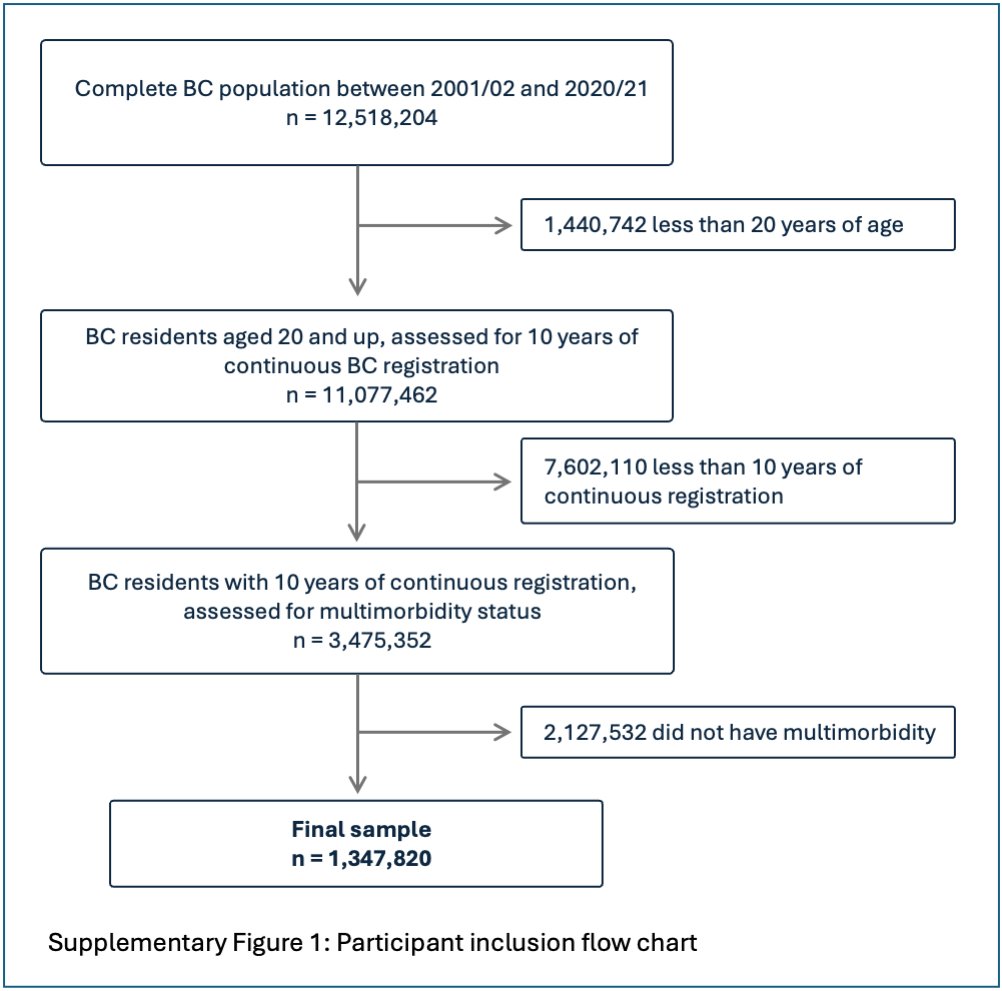


**Figure S1: Participant inclusion flow chart**


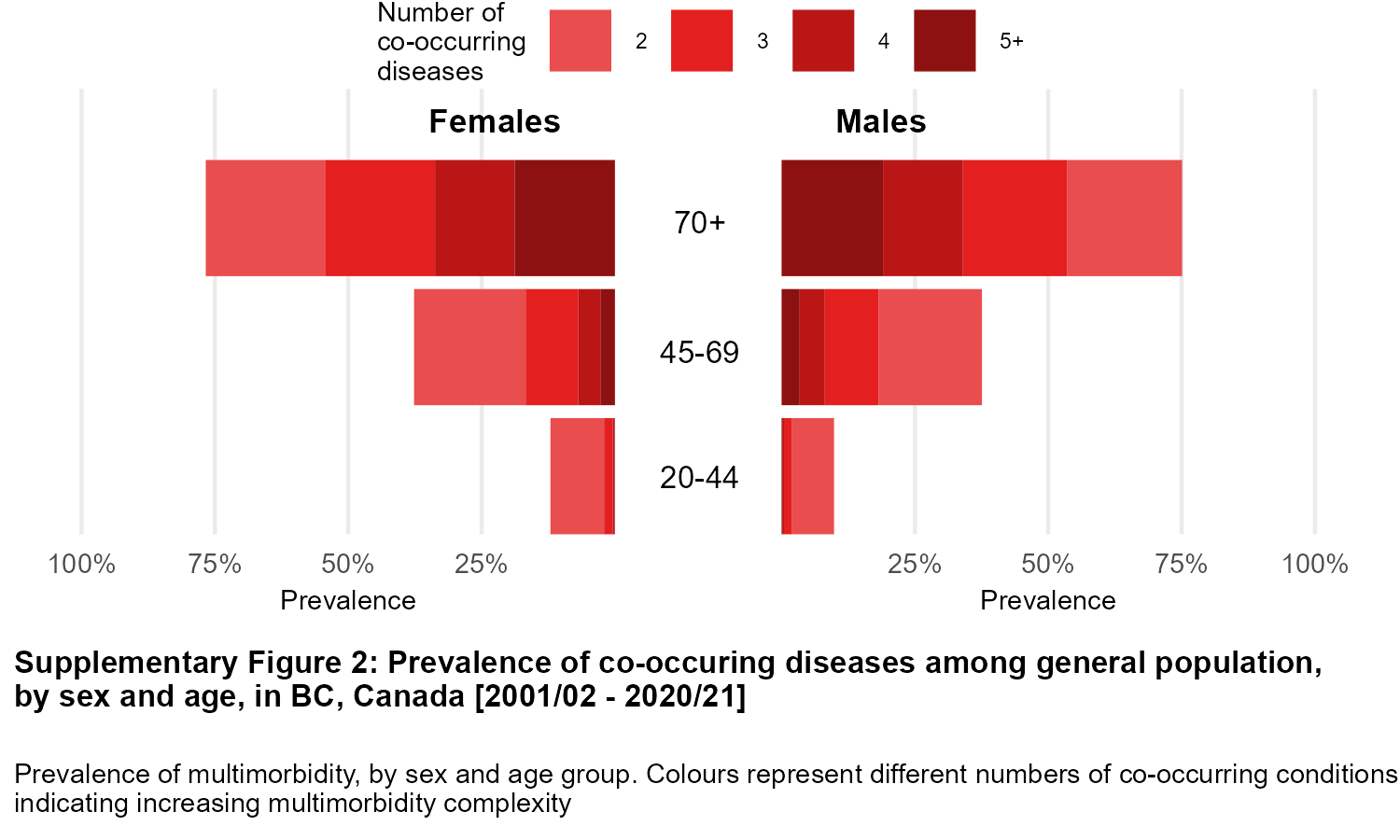


**Figure S2: Prevalence of co-occurring diseases among general population, by sex and age, in BC, Canada [2001/02 - 2020/21]**

Prevalence of multimorbidity, by sex and age group. Colours represent different numbers of co-occurring conditions indicating increasing multimorbidity complexity

| Table S1: Directed link data for top 15 most prevalent network links | | |
| --- | --- | --- |
| Disease Nodes | Prevalence (per 100) | Lift |
| **20-44** | | |
| **Females** | | |
| Asthma ➞ Mood & anxiety disorders | 3.27 | 2.94 |
| Mood & anxiety disorders ➞ Hypertension | 1.07 | 2.11 |
| Mood & anxiety disorders ➞ Diabetes | 1.02 | 2.11 |
| Mood & anxiety disorders ➞ Schizophrenia | 0.68 | 2.70 |
| Mood & anxiety disorders ➞ Osteoarthritis | 0.47 | 2.12 |
| Mood & anxiety disorders ➞ Chronic kidney disease | 0.34 | 1.89 |
| Mood & anxiety disorders ➞ Epilepsy | 0.27 | 2.21 |
| Mood & anxiety disorders ➞ Rheumatoid arthritis | 0.25 | 2.04 |
| Mood & anxiety disorders ➞ Heart disease | 0.21 | 1.97 |
| Mood & anxiety disorders ➞ COPD | 0.21 | 1.69 |
| Asthma ➞ COPD | 0.18 | 2.12 |
| Mood & anxiety disorders ➞ Heart failure | 0.10 | 1.78 |
| Mood & anxiety disorders ➞ Multiple sclerosis | 0.09 | 2.38 |
| Diabetes ➞ Chronic kidney disease | 0.09 | 3.51 |
| Asthma ➞ Rheumatoid arthritis | 0.09 | 1.04 |
| **Males** | | |
| Asthma ➞ Mood & anxiety disorders | 1.99 | 3.18 |
| Mood & anxiety disorders ➞ Schizophrenia | 1.26 | 3.17 |
| Mood & anxiety disorders ➞ Hypertension | 1.00 | 2.07 |
| Mood & anxiety disorders ➞ Diabetes | 0.52 | 1.85 |
| Mood & anxiety disorders ➞ Osteoarthritis | 0.32 | 2.25 |
| Mood & anxiety disorders ➞ Chronic kidney disease | 0.26 | 1.76 |
| Mood & anxiety disorders ➞ Gout | 0.25 | 1.83 |
| Mood & anxiety disorders ➞ Heart disease | 0.23 | 1.96 |
| Mood & anxiety disorders ➞ COPD | 0.18 | 1.89 |
| Asthma ➞ Diabetes | 0.18 | 1.06 |
| Asthma ➞ COPD | 0.13 | 2.36 |
| Diabetes ➞ Chronic kidney disease | 0.10 | 4.19 |
| Mood & anxiety disorders ➞ Heart failure | 0.10 | 1.71 |
| Schizophrenia ➞ Diabetes | 0.10 | 2.90 |
| Asthma ➞ Osteoarthritis | 0.09 | 1.07 |
| **45-69** | | |
| **Females** | | |
| Mood & anxiety disorders ➞ Hypertension | 4.52 | 3.52 |
| Hypertension ➞ Diabetes | 3.56 | 4.29 |
| Mood & anxiety disorders ➞ Osteoarthritis | 3.17 | 3.30 |
| Hypertension ➞ Osteoarthritis | 3.01 | 3.23 |
| Mood & anxiety disorders ➞ Diabetes | 2.43 | 2.85 |
| Mood & anxiety disorders ➞ Asthma | 2.10 | 3.73 |
| Hypertension ➞ Heart disease | 1.95 | 4.01 |
| Asthma ➞ Hypertension | 1.76 | 3.04 |
| Mood & anxiety disorders ➞ Osteoporosis | 1.38 | 2.94 |
| Hypertension ➞ Chronic kidney disease | 1.36 | 4.05 |
| Mood & anxiety disorders ➞ Heart disease | 1.33 | 2.65 |
| Asthma ➞ Osteoarthritis | 1.33 | 3.06 |
| Hypertension ➞ Osteoporosis | 1.30 | 2.86 |
| Hypertension ➞ COPD | 1.24 | 3.06 |
| Asthma ➞ COPD | 1.15 | 6.09 |
| **Males** | | |
| Mood & anxiety disorders ➞ Hypertension | 4.69 | 3.75 |
| Hypertension ➞ Diabetes | 4.66 | 4.45 |
| Hypertension ➞ Heart disease | 3.36 | 4.01 |
| Hypertension ➞ Osteoarthritis | 2.70 | 3.53 |
| Mood & anxiety disorders ➞ Diabetes | 2.50 | 3.10 |
| Mood & anxiety disorders ➞ Osteoarthritis | 2.30 | 3.89 |
| Mood & anxiety disorders ➞ Heart disease | 2.03 | 3.14 |
| Hypertension ➞ Chronic kidney disease | 1.89 | 4.24 |
| Diabetes ➞ Heart disease | 1.78 | 4.17 |
| Hypertension ➞ Gout | 1.75 | 4.36 |
| Hypertension ➞ COPD | 1.60 | 3.27 |
| Asthma ➞ Hypertension | 1.44 | 3.16 |
| Hypertension ➞ Heart failure | 1.34 | 4.06 |
| Mood & anxiety disorders ➞ Asthma | 1.34 | 4.12 |
| Mood & anxiety disorders ➞ COPD | 1.30 | 3.45 |
| **70+** | | |
| **Females** | | |
| Hypertension ➞ Osteoarthritis | 9.06 | 6.11 |
| Hypertension ➞ Chronic kidney disease | 8.10 | 5.99 |
| Hypertension ➞ Heart disease | 7.28 | 6.21 |
| Hypertension ➞ Osteoporosis | 6.86 | 5.80 |
| Hypertension ➞ Mood & anxiety disorders | 6.74 | 6.00 |
| Hypertension ➞ Diabetes | 6.57 | 6.58 |
| Hypertension ➞ Heart failure | 6.47 | 5.52 |
| Osteoporosis ➞ Osteoarthritis | 5.72 | 5.87 |
| Hypertension ➞ Alzheimer's & dementia | 5.48 | 5.44 |
| Osteoarthritis ➞ Chronic kidney disease | 5.31 | 5.80 |
| Hypertension ➞ COPD | 5.29 | 5.71 |
| Heart disease ➞ Heart failure | 5.11 | 9.19 |
| Osteoporosis ➞ Chronic kidney disease | 4.94 | 5.56 |
| Mood & anxiety disorders ➞ Osteoarthritis | 4.86 | 5.69 |
| Heart disease ➞ Chronic kidney disease | 4.65 | 7.24 |
| **Males** | | |
| Hypertension ➞ Chronic kidney disease | 9.94 | 6.14 |
| Hypertension ➞ Heart disease | 9.87 | 6.23 |
| Hypertension ➞ Diabetes | 8.68 | 6.64 |
| Hypertension ➞ Heart failure | 8.27 | 5.66 |
| Hypertension ➞ Osteoarthritis | 7.92 | 6.23 |
| Heart disease ➞ Heart failure | 7.40 | 8.45 |
| Hypertension ➞ COPD | 6.78 | 5.85 |
| Hypertension ➞ Mood & anxiety disorders | 6.51 | 5.96 |
| Heart disease ➞ Chronic kidney disease | 6.49 | 6.69 |
| Diabetes ➞ Chronic kidney disease | 6.00 | 7.08 |
| Heart failure ➞ Chronic kidney disease | 5.11 | 8.55 |
| Hypertension ➞ Alzheimer's & dementia | 5.04 | 5.53 |
| Diabetes ➞ Heart disease | 4.98 | 6.01 |
| Osteoarthritis ➞ Chronic kidney disease | 4.52 | 5.69 |
| Diabetes ➞ Heart failure | 4.47 | 5.85 |
| The top 15 most prevalent directed links for each age- and sex-stratified network | | |

| Table S2: Network descriptive statistics | | | | | | | |  |
| --- | --- | --- | --- | --- | --- | --- | --- | --- |
| Network |  |  | Network density | In degree (to node) | Out degree (from node) | In strength (to node) | Out strength (from node) | |
| **20-44** | | | | | | | |  |
| **Females** |  |  | 0.063 |  |  |  |  | |
|  | Disease node | COPD |  | 5 | 0 | 0.507 | 0.000 | |
|  |  | Heart disease |  | 3 | 0 | 0.318 | 0.000 | |
|  |  | Other solid cancer |  | 2 | 0 | 0.100 | 0.000 | |
|  |  | Heart failure |  | 2 | 0 | 0.102 | 0.000 | |
|  |  | Hypertension |  | 2 | 3 | 1.111 | 0.144 | |
|  |  | Diabetes |  | 2 | 3 | 1.082 | 0.179 | |
|  |  | Rheumatoid arthritis |  | 2 | 0 | 0.332 | 0.000 | |
|  |  | Chronic kidney disease |  | 2 | 0 | 0.430 | 0.000 | |
|  |  | Breast cancer |  | 1 | 1 | 0.056 | 0.004 | |
|  |  | Colorectal cancer |  | 1 | 0 | 0.018 | 0.000 | |
|  |  | Haematological cancer |  | 1 | 0 | 0.023 | 0.000 | |
|  |  | Melanoma cancer |  | 1 | 0 | 0.013 | 0.000 | |
|  |  | Stroke |  | 1 | 0 | 0.038 | 0.000 | |
|  |  | Mood & anxiety disorders |  | 1 | 19 | 3.274 | 5.040 | |
|  |  | Schizophrenia |  | 1 | 3 | 0.682 | 0.118 | |
|  |  | Gout |  | 1 | 0 | 0.079 | 0.000 | |
|  |  | Osteoarthritis |  | 1 | 0 | 0.470 | 0.000 | |
|  |  | Alzheimer's & dementia |  | 1 | 0 | 0.021 | 0.000 | |
|  |  | Epilepsy |  | 1 | 0 | 0.270 | 0.000 | |
|  |  | Multiple sclerosis |  | 1 | 0 | 0.095 | 0.000 | |
|  |  | Asthma |  | 0 | 3 | 0.000 | 3.537 | |
|  |  | Lung cancer |  | 0 | 0 | 0.000 | 0.000 | |
|  |  | Parkinsonism |  | 0 | 0 | 0.000 | 0.000 | |
| **Males** |  |  | 0.075 |  |  |  |  | |
|  | Disease node | COPD |  | 5 | 0 | 0.415 | 0.000 | |
|  |  | Diabetes |  | 4 | 2 | 0.866 | 0.159 | |
|  |  | Other solid cancer |  | 3 | 0 | 0.086 | 0.000 | |
|  |  | Heart disease |  | 3 | 0 | 0.318 | 0.000 | |
|  |  | Rheumatoid arthritis |  | 3 | 0 | 0.094 | 0.000 | |
|  |  | Chronic kidney disease |  | 3 | 0 | 0.389 | 0.000 | |
|  |  | Haematological cancer |  | 2 | 0 | 0.024 | 0.000 | |
|  |  | Heart failure |  | 2 | 0 | 0.110 | 0.000 | |
|  |  | Hypertension |  | 2 | 2 | 1.084 | 0.077 | |
|  |  | Gout |  | 2 | 2 | 0.335 | 0.089 | |
|  |  | Osteoarthritis |  | 2 | 0 | 0.414 | 0.000 | |
|  |  | Colorectal cancer |  | 1 | 0 | 0.003 | 0.000 | |
|  |  | Stroke |  | 1 | 0 | 0.033 | 0.000 | |
|  |  | Mood & anxiety disorders |  | 1 | 15 | 1.992 | 4.318 | |
|  |  | Schizophrenia |  | 1 | 6 | 1.256 | 0.278 | |
|  |  | Alzheimer's & dementia |  | 1 | 0 | 0.017 | 0.000 | |
|  |  | Epilepsy |  | 1 | 1 | 0.071 | 0.013 | |
|  |  | Multiple sclerosis |  | 1 | 0 | 0.030 | 0.000 | |
|  |  | Asthma |  | 0 | 10 | 0.000 | 2.604 | |
|  |  | Lung cancer |  | 0 | 0 | 0.000 | 0.000 | |
|  |  | Melanoma cancer |  | 0 | 0 | 0.000 | 0.000 | |
|  |  | Prostate cancer |  | 0 | 0 | 0.000 | 0.000 | |
|  |  | Parkinsonism |  | 0 | 0 | 0.000 | 0.000 | |
| **45-69** | | | | | | | |  |
| **Females** |  |  | 0.310 |  |  |  |  | |
|  | Disease node | Lung cancer |  | 14 | 0 | 0.936 | 0.000 | |
|  |  | Other solid cancer |  | 14 | 0 | 1.867 | 0.000 | |
|  |  | Alzheimer's & dementia |  | 14 | 0 | 0.771 | 0.000 | |
|  |  | Heart failure |  | 12 | 1 | 3.117 | 0.040 | |
|  |  | Breast cancer |  | 11 | 4 | 1.210 | 0.188 | |
|  |  | Chronic kidney disease |  | 11 | 2 | 5.296 | 0.078 | |
|  |  | Haematological cancer |  | 10 | 0 | 0.329 | 0.000 | |
|  |  | Stroke |  | 10 | 2 | 1.024 | 0.061 | |
|  |  | Colorectal cancer |  | 9 | 0 | 0.381 | 0.000 | |
|  |  | Heart disease |  | 9 | 9 | 6.424 | 1.302 | |
|  |  | Osteoporosis |  | 9 | 9 | 5.178 | 1.287 | |
|  |  | COPD |  | 9 | 9 | 5.095 | 1.045 | |
|  |  | Melanoma cancer |  | 6 | 0 | 0.129 | 0.000 | |
|  |  | Gout |  | 6 | 5 | 1.435 | 0.070 | |
|  |  | Parkinsonism |  | 6 | 1 | 0.168 | 0.015 | |
|  |  | Osteoarthritis |  | 5 | 16 | 7.653 | 5.118 | |
|  |  | Diabetes |  | 4 | 15 | 7.279 | 4.252 | |
|  |  | Rheumatoid arthritis |  | 4 | 12 | 1.656 | 0.758 | |
|  |  | Epilepsy |  | 3 | 1 | 0.344 | 0.060 | |
|  |  | Hypertension |  | 2 | 19 | 6.284 | 15.731 | |
|  |  | Schizophrenia |  | 1 | 14 | 0.314 | 0.661 | |
|  |  | Multiple sclerosis |  | 1 | 10 | 0.115 | 0.194 | |
|  |  | Asthma |  | 1 | 19 | 2.095 | 8.246 | |
|  |  | Mood & anxiety disorders |  | 0 | 23 | 0.000 | 19.992 | |
| **Males** |  |  | 0.304 |  |  |  |  | |
|  | Disease node | Lung cancer |  | 14 | 0 | 0.886 | 0.000 | |
|  |  | Chronic kidney disease |  | 14 | 1 | 7.390 | 0.067 | |
|  |  | Alzheimer's & dementia |  | 14 | 0 | 1.076 | 0.000 | |
|  |  | Other solid cancer |  | 13 | 2 | 2.291 | 0.163 | |
|  |  | Colorectal cancer |  | 10 | 2 | 0.559 | 0.048 | |
|  |  | Osteoporosis |  | 10 | 1 | 1.766 | 0.046 | |
|  |  | COPD |  | 10 | 9 | 6.622 | 1.054 | |
|  |  | Haematological cancer |  | 9 | 0 | 0.475 | 0.000 | |
|  |  | Heart failure |  | 9 | 4 | 4.995 | 0.650 | |
|  |  | Melanoma cancer |  | 8 | 0 | 0.163 | 0.000 | |
|  |  | Prostate cancer |  | 8 | 1 | 0.992 | 0.008 | |
|  |  | Stroke |  | 8 | 2 | 1.530 | 0.113 | |
|  |  | Heart disease |  | 7 | 12 | 9.552 | 3.677 | |
|  |  | Parkinsonism |  | 7 | 2 | 0.302 | 0.037 | |
|  |  | Diabetes |  | 5 | 14 | 9.104 | 5.851 | |
|  |  | Rheumatoid arthritis |  | 5 | 8 | 0.881 | 0.269 | |
|  |  | Epilepsy |  | 5 | 2 | 0.657 | 0.113 | |
|  |  | Osteoarthritis |  | 4 | 15 | 5.790 | 4.036 | |
|  |  | Hypertension |  | 3 | 19 | 6.328 | 20.415 | |
|  |  | Gout |  | 3 | 17 | 2.991 | 2.760 | |
|  |  | Schizophrenia |  | 1 | 13 | 0.482 | 0.899 | |
|  |  | Asthma |  | 1 | 20 | 1.338 | 6.436 | |
|  |  | Mood & anxiety disorders |  | 0 | 22 | 0.000 | 19.508 | |
|  |  | Multiple sclerosis |  | 0 | 2 | 0.000 | 0.018 | |
| **70+** | | | | | | | |  |
| **Females** |  |  | 0.370 |  |  |  |  | |
|  | Disease node | Lung cancer |  | 19 | 0 | 5.298 | 0.000 | |
|  |  | Other solid cancer |  | 19 | 1 | 8.626 | 0.045 | |
|  |  | Alzheimer's & dementia |  | 15 | 2 | 34.533 | 0.397 | |
|  |  | Breast cancer |  | 14 | 2 | 4.216 | 0.096 | |
|  |  | Colorectal cancer |  | 14 | 1 | 2.920 | 0.020 | |
|  |  | Chronic kidney disease |  | 14 | 7 | 43.215 | 4.310 | |
|  |  | Heart failure |  | 13 | 9 | 33.964 | 9.712 | |
|  |  | Haematological cancer |  | 12 | 1 | 1.942 | 0.013 | |
|  |  | Melanoma cancer |  | 12 | 1 | 0.623 | 0.011 | |
|  |  | Stroke |  | 11 | 3 | 10.783 | 1.208 | |
|  |  | Epilepsy |  | 10 | 0 | 1.661 | 0.000 | |
|  |  | Parkinsonism |  | 9 | 7 | 2.334 | 0.884 | |
|  |  | COPD |  | 8 | 10 | 23.055 | 9.983 | |
|  |  | Heart disease |  | 7 | 14 | 24.899 | 19.899 | |
|  |  | Gout |  | 7 | 12 | 7.563 | 3.846 | |
|  |  | Rheumatoid arthritis |  | 4 | 14 | 3.410 | 3.173 | |
|  |  | Schizophrenia |  | 4 | 6 | 0.838 | 0.350 | |
|  |  | Diabetes |  | 3 | 15 | 12.458 | 20.214 | |
|  |  | Osteoarthritis |  | 3 | 17 | 19.642 | 28.971 | |
|  |  | Asthma |  | 3 | 15 | 6.805 | 11.437 | |
|  |  | Mood & anxiety disorders |  | 2 | 20 | 11.056 | 33.531 | |
|  |  | Osteoporosis |  | 1 | 21 | 6.860 | 43.213 | |
|  |  | Hypertension |  | 0 | 22 | 0.000 | 75.310 | |
|  |  | Multiple sclerosis |  | 0 | 4 | 0.000 | 0.079 | |
| **Males** |  |  | 0.357 |  |  |  |  | |
|  | Disease node | Lung cancer |  | 21 | 0 | 5.923 | 0.000 | |
|  |  | Other solid cancer |  | 17 | 1 | 11.785 | 0.084 | |
|  |  | Alzheimer's & dementia |  | 17 | 2 | 29.475 | 0.437 | |
|  |  | Haematological cancer |  | 15 | 1 | 3.111 | 0.020 | |
|  |  | Chronic kidney disease |  | 13 | 6 | 45.449 | 4.535 | |
|  |  | Colorectal cancer |  | 12 | 1 | 3.239 | 0.030 | |
|  |  | Melanoma cancer |  | 12 | 1 | 1.093 | 0.025 | |
|  |  | Prostate cancer |  | 12 | 3 | 6.273 | 0.301 | |
|  |  | Heart failure |  | 12 | 8 | 36.914 | 9.932 | |
|  |  | Stroke |  | 10 | 4 | 9.780 | 1.476 | |
|  |  | Parkinsonism |  | 9 | 6 | 3.670 | 1.260 | |
|  |  | COPD |  | 9 | 11 | 24.339 | 12.591 | |
|  |  | Epilepsy |  | 8 | 3 | 1.837 | 0.270 | |
|  |  | Osteoporosis |  | 7 | 9 | 8.463 | 5.090 | |
|  |  | Gout |  | 6 | 15 | 12.131 | 9.736 | |
|  |  | Rheumatoid arthritis |  | 4 | 12 | 1.877 | 1.844 | |
|  |  | Heart disease |  | 3 | 16 | 19.201 | 30.680 | |
|  |  | Schizophrenia |  | 3 | 6 | 0.608 | 0.425 | |
|  |  | Osteoarthritis |  | 3 | 17 | 14.813 | 27.957 | |
|  |  | Asthma |  | 2 | 15 | 4.012 | 9.376 | |
|  |  | Diabetes |  | 1 | 18 | 8.680 | 33.977 | |
|  |  | Mood & anxiety disorders |  | 1 | 18 | 6.514 | 25.239 | |
|  |  | Hypertension |  | 0 | 22 | 0.000 | 83.869 | |
|  |  | Multiple sclerosis |  | 0 | 2 | 0.000 | 0.032 | |
| Network summary statistics for each age- and sex-stratified directed network. Degree is the number of links coming into (to node) or out from (from node) each disease node. Strength is a sum of the link weights for all links coming into (to node) or out from (from node) each disease node | | | | | | | |  |

| Table S3: Directed link data for top 15 highest lift network links | | |
| --- | --- | --- |
| Disease Nodes | Lift | Prevalence (per 100) |
| **20-44** | | |
| **Females** | | |
| Breast cancer ➞ Heart failure | 12.08 | 0.00 |
| Hypertension ➞ Heart disease | 3.88 | 0.07 |
| Diabetes ➞ Chronic kidney disease | 3.51 | 0.09 |
| Schizophrenia ➞ COPD | 3.45 | 0.02 |
| Hypertension ➞ COPD | 3.25 | 0.06 |
| Schizophrenia ➞ Diabetes | 3.15 | 0.06 |
| Diabetes ➞ Heart disease | 3.07 | 0.05 |
| Asthma ➞ Mood & anxiety disorders | 2.94 | 3.27 |
| Mood & anxiety disorders ➞ Schizophrenia | 2.70 | 0.68 |
| Diabetes ➞ COPD | 2.60 | 0.04 |
| Hypertension ➞ Other solid cancer | 2.45 | 0.02 |
| Mood & anxiety disorders ➞ Melanoma cancer | 2.45 | 0.01 |
| Mood & anxiety disorders ➞ Colorectal cancer | 2.38 | 0.02 |
| Mood & anxiety disorders ➞ Multiple sclerosis | 2.38 | 0.09 |
| Mood & anxiety disorders ➞ Breast cancer | 2.28 | 0.06 |
| **Males** | | |
| Gout ➞ Rheumatoid arthritis | 6.29 | 0.01 |
| Diabetes ➞ Chronic kidney disease | 4.19 | 0.10 |
| Gout ➞ Diabetes | 3.58 | 0.07 |
| Diabetes ➞ Heart disease | 3.22 | 0.06 |
| Asthma ➞ Mood & anxiety disorders | 3.18 | 1.99 |
| Mood & anxiety disorders ➞ Schizophrenia | 3.17 | 1.26 |
| Schizophrenia ➞ Diabetes | 2.90 | 0.10 |
| Hypertension ➞ COPD | 2.60 | 0.06 |
| Mood & anxiety disorders ➞ Multiple sclerosis | 2.50 | 0.03 |
| Asthma ➞ COPD | 2.36 | 0.13 |
| Mood & anxiety disorders ➞ Alzheimer's & dementia | 2.32 | 0.02 |
| Mood & anxiety disorders ➞ Osteoarthritis | 2.25 | 0.32 |
| Schizophrenia ➞ COPD | 2.10 | 0.02 |
| Mood & anxiety disorders ➞ Other solid cancer | 2.10 | 0.06 |
| Mood & anxiety disorders ➞ Hypertension | 2.07 | 1.00 |
| **45-69** | | |
| **Females** | | |
| Parkinsonism ➞ Alzheimer's & dementia | 55.44 | 0.02 |
| Stroke ➞ Epilepsy | 22.85 | 0.04 |
| Schizophrenia ➞ Alzheimer's & dementia | 18.77 | 0.04 |
| Stroke ➞ Alzheimer's & dementia | 16.73 | 0.02 |
| Schizophrenia ➞ Parkinsonism | 12.62 | 0.01 |
| COPD ➞ Lung cancer | 12.17 | 0.14 |
| Heart disease ➞ Heart failure | 10.72 | 0.48 |
| Multiple sclerosis ➞ Alzheimer's & dementia | 10.69 | 0.01 |
| Breast cancer ➞ Osteoporosis | 8.66 | 0.13 |
| COPD ➞ Heart failure | 7.55 | 0.28 |
| Chronic kidney disease ➞ Alzheimer's & dementia | 7.23 | 0.04 |
| Osteoporosis ➞ Alzheimer's & dementia | 6.26 | 0.07 |
| Asthma ➞ COPD | 6.09 | 1.15 |
| Heart disease ➞ Stroke | 5.87 | 0.10 |
| Osteoarthritis ➞ Rheumatoid arthritis | 5.86 | 0.51 |
| **Males** | | |
| Parkinsonism ➞ Alzheimer's & dementia | 63.28 | 0.03 |
| Stroke ➞ Epilepsy | 19.80 | 0.07 |
| Schizophrenia ➞ Alzheimer's & dementia | 18.34 | 0.05 |
| Stroke ➞ Alzheimer's & dementia | 18.02 | 0.05 |
| Multiple sclerosis ➞ Alzheimer's & dementia | 16.66 | 0.01 |
| COPD ➞ Lung cancer | 13.30 | 0.15 |
| Rheumatoid arthritis ➞ Osteoporosis | 9.45 | 0.05 |
| Heart disease ➞ Heart failure | 8.78 | 1.21 |
| Other solid cancer ➞ Chronic kidney disease | 8.58 | 0.15 |
| Other solid cancer ➞ Lung cancer | 8.54 | 0.02 |
| Heart failure ➞ Chronic kidney disease | 8.51 | 0.48 |
| Prostate cancer ➞ Lung cancer | 8.50 | 0.01 |
| Colorectal cancer ➞ Chronic kidney disease | 8.12 | 0.04 |
| Chronic kidney disease ➞ Alzheimer's & dementia | 7.95 | 0.07 |
| Osteoarthritis ➞ Rheumatoid arthritis | 7.63 | 0.25 |
| **70+** | | |
| **Females** | | |
| Stroke ➞ Epilepsy | 23.88 | 0.13 |
| COPD ➞ Lung cancer | 15.69 | 0.71 |
| Parkinsonism ➞ Alzheimer's & dementia | 13.89 | 0.42 |
| Asthma ➞ COPD | 11.52 | 2.69 |
| Melanoma cancer ➞ Other solid cancer | 10.83 | 0.01 |
| Stroke ➞ Alzheimer's & dementia | 10.49 | 0.95 |
| Breast cancer ➞ Lung cancer | 9.32 | 0.04 |
| Heart disease ➞ Heart failure | 9.19 | 5.11 |
| Heart failure ➞ Stroke | 9.16 | 1.15 |
| Heart failure ➞ Chronic kidney disease | 9.02 | 4.32 |
| Gout ➞ Chronic kidney disease | 8.81 | 1.35 |
| Mood & anxiety disorders ➞ Schizophrenia | 8.62 | 0.26 |
| Other solid cancer ➞ Lung cancer | 8.53 | 0.05 |
| Colorectal cancer ➞ Lung cancer | 8.30 | 0.02 |
| Osteoarthritis ➞ Rheumatoid arthritis | 8.16 | 1.05 |
| **Males** | | |
| Stroke ➞ Epilepsy | 20.65 | 0.17 |
| Parkinsonism ➞ Alzheimer's & dementia | 18.68 | 0.77 |
| COPD ➞ Lung cancer | 13.93 | 0.82 |
| Mood & anxiety disorders ➞ Schizophrenia | 11.86 | 0.24 |
| Stroke ➞ Alzheimer's & dementia | 11.14 | 1.00 |
| Asthma ➞ COPD | 11.08 | 2.34 |
| Epilepsy ➞ Alzheimer's & dementia | 10.48 | 0.22 |
| Melanoma cancer ➞ Other solid cancer | 9.98 | 0.03 |
| Osteoarthritis ➞ Rheumatoid arthritis | 9.76 | 0.62 |
| Other solid cancer ➞ Lung cancer | 8.96 | 0.08 |
| Osteoporosis ➞ Alzheimer's & dementia | 8.69 | 1.17 |
| Mood & anxiety disorders ➞ Alzheimer's & dementia | 8.59 | 3.45 |
| Heart failure ➞ Chronic kidney disease | 8.55 | 5.11 |
| Heart disease ➞ Heart failure | 8.45 | 7.40 |
| Asthma ➞ Lung cancer | 8.41 | 0.26 |
| The top 15 highest lift links for each age- and sex-stratified network | | |

| Table S4: Lift network clusters | |
| --- | --- |
| **20-44** | |
| **Males** | |
| Number of clusters: 3 | |
| Cluster 1: Mood & anxiety disorders | |
| Cluster prevalence (per 100) | 0.018 |
| Average link weight in cluster | 1.859 |
| Cluster links | Mood & anxiety disorders ➞ Chronic kidney disease |
|  | Mood & anxiety disorders ➞ Heart disease |
| Cluster 2: Diabetes | |
| Cluster prevalence (per 100) | 0.011 |
| Average link weight in cluster | 3.704 |
| Cluster links | Diabetes ➞ Chronic kidney disease |
|  | Diabetes ➞ Heart disease |
| Cluster 3: Schizophrenia | |
| Cluster prevalence (per 100) | 0.003 |
| Average link weight in cluster | 1.775 |
| Cluster links | Schizophrenia ➞ Chronic kidney disease |
|  | Schizophrenia ➞ Heart disease |
| **45-69** | |
| **Females** | |
| Number of clusters: 27 | |
| Cluster 1: Diabetes | |
| Cluster prevalence (per 100) | 0.428 |
| Average link weight in cluster | 3.63 |
| Cluster links | Asthma ➞ Diabetes |
|  | Hypertension ➞ Diabetes |
| Cluster 2: Asthma + Hypertension + Osteoarthritis | |
| Cluster prevalence (per 100) | 0.193 |
| Average link weight in cluster | 3.109 |
| Cluster links | Asthma ➞ Hypertension |
|  | Asthma ➞ Osteoarthritis |
|  | Hypertension ➞ Osteoarthritis |
| Cluster 3: Mood & anxiety disorders | |
| Cluster prevalence (per 100) | 0.042 |
| Average link weight in cluster | 3.35 |
| Cluster links | Mood & anxiety disorders ➞ Asthma |
|  | Mood & anxiety disorders ➞ Diabetes |
|  | Mood & anxiety disorders ➞ Hypertension |
|  | Mood & anxiety disorders ➞ Osteoarthritis |
| Cluster 4: Other solid cancer | |
| Cluster prevalence (per 100) | 0.017 |
| Average link weight in cluster | 3.949 |
| Cluster links | COPD ➞ Other solid cancer |
|  | Osteoporosis ➞ Other solid cancer |
| Cluster 5: Chronic kidney disease | |
| Cluster prevalence (per 100) | 0.016 |
| Average link weight in cluster | 4.425 |
| Cluster links | COPD ➞ Chronic kidney disease |
|  | Heart disease ➞ Chronic kidney disease |
|  | Osteoporosis ➞ Chronic kidney disease |
| Cluster 6: Heart failure | |
| Cluster prevalence (per 100) | 0.016 |
| Average link weight in cluster | 7.517 |
| Cluster links | COPD ➞ Heart failure |
|  | Heart disease ➞ Heart failure |
|  | Osteoporosis ➞ Heart failure |
| Cluster 7: Colorectal cancer | |
| Cluster prevalence (per 100) | 0.013 |
| Average link weight in cluster | 3.107 |
| Cluster links | Asthma ➞ Colorectal cancer |
|  | Hypertension ➞ Colorectal cancer |
| Cluster 8: Multiple sclerosis | |
| Cluster prevalence (per 100) | 0.009 |
| Average link weight in cluster | 3.37 |
| Cluster links | Mood & anxiety disorders ➞ Multiple sclerosis |
|  | Multiple sclerosis ➞ Osteoarthritis |
| Cluster 9: Haematological cancer | |
| Cluster prevalence (per 100) | 0.009 |
| Average link weight in cluster | 2.73 |
| Cluster links | Asthma ➞ Haematological cancer |
|  | Hypertension ➞ Haematological cancer |
| Cluster 10: COPD | |
| Cluster prevalence (per 100) | 0.008 |
| Average link weight in cluster | 3.645 |
| Cluster links | Asthma ➞ COPD |
|  | Diabetes ➞ COPD |
|  | Hypertension ➞ COPD |
|  | Mood & anxiety disorders ➞ COPD |
|  | Osteoarthritis ➞ COPD |
| Cluster 11: Rheumatoid arthritis | |
| Cluster prevalence (per 100) | 0.008 |
| Average link weight in cluster | 3.589 |
| Cluster links | Asthma ➞ Rheumatoid arthritis |
|  | Hypertension ➞ Rheumatoid arthritis |
|  | Mood & anxiety disorders ➞ Rheumatoid arthritis |
|  | Osteoarthritis ➞ Rheumatoid arthritis |
| Cluster 12: Breast cancer | |
| Cluster prevalence (per 100) | 0.006 |
| Average link weight in cluster | 5.603 |
| Cluster links | Breast cancer ➞ Osteoporosis |
|  | COPD ➞ Breast cancer |
| Cluster 13: Rheumatoid arthritis | |
| Cluster prevalence (per 100) | 0.006 |
| Average link weight in cluster | 3.81 |
| Cluster links | Rheumatoid arthritis ➞ COPD |
|  | Rheumatoid arthritis ➞ Heart disease |
|  | Rheumatoid arthritis ➞ Osteoporosis |
| Cluster 14: Lung cancer | |
| Cluster prevalence (per 100) | 0.005 |
| Average link weight in cluster | 7.463 |
| Cluster links | COPD ➞ Lung cancer |
|  | Heart disease ➞ Lung cancer |
|  | Osteoporosis ➞ Lung cancer |
| Cluster 15: Heart failure | |
| Cluster prevalence (per 100) | 0.005 |
| Average link weight in cluster | 3.443 |
| Cluster links | Asthma ➞ Heart failure |
|  | Diabetes ➞ Heart failure |
|  | Hypertension ➞ Heart failure |
|  | Mood & anxiety disorders ➞ Heart failure |
|  | Osteoarthritis ➞ Heart failure |
| Cluster 16: Alzheimer's & dementia | |
| Cluster prevalence (per 100) | 0.005 |
| Average link weight in cluster | 5.374 |
| Cluster links | COPD ➞ Alzheimer's & dementia |
|  | Heart disease ➞ Alzheimer's & dementia |
|  | Osteoporosis ➞ Alzheimer's & dementia |
| Cluster 17: Osteoporosis | |
| Cluster prevalence (per 100) | 0.005 |
| Average link weight in cluster | 2.967 |
| Cluster links | Asthma ➞ Osteoporosis |
|  | Diabetes ➞ Osteoporosis |
|  | Hypertension ➞ Osteoporosis |
|  | Mood & anxiety disorders ➞ Osteoporosis |
|  | Osteoarthritis ➞ Osteoporosis |
| Cluster 18: Schizophrenia | |
| Cluster prevalence (per 100) | 0.004 |
| Average link weight in cluster | 3.587 |
| Cluster links | Mood & anxiety disorders ➞ Schizophrenia |
|  | Schizophrenia ➞ Diabetes |
|  | Schizophrenia ➞ Osteoarthritis |
| Cluster 19: Stroke | |
| Cluster prevalence (per 100) | 0.003 |
| Average link weight in cluster | 4.936 |
| Cluster links | COPD ➞ Stroke |
|  | Heart disease ➞ Stroke |
|  | Osteoporosis ➞ Stroke |
| Cluster 20: Gout | |
| Cluster prevalence (per 100) | 0.002 |
| Average link weight in cluster | 3.518 |
| Cluster links | Asthma ➞ Gout |
|  | Diabetes ➞ Gout |
|  | Hypertension ➞ Gout |
|  | Mood & anxiety disorders ➞ Gout |
|  | Osteoarthritis ➞ Gout |
| Cluster 21: Schizophrenia | |
| Cluster prevalence (per 100) | 0.002 |
| Average link weight in cluster | 3.452 |
| Cluster links | Schizophrenia ➞ COPD |
|  | Schizophrenia ➞ Heart disease |
|  | Schizophrenia ➞ Osteoporosis |
| Cluster 22: Epilepsy | |
| Cluster prevalence (per 100) | 0.002 |
| Average link weight in cluster | 3.454 |
| Cluster links | Epilepsy ➞ COPD |
|  | Hypertension ➞ Epilepsy |
|  | Mood & anxiety disorders ➞ Epilepsy |
| Cluster 23: Other solid cancer | |
| Cluster prevalence (per 100) | 0.002 |
| Average link weight in cluster | 3.244 |
| Cluster links | Asthma ➞ Other solid cancer |
|  | Diabetes ➞ Other solid cancer |
|  | Hypertension ➞ Other solid cancer |
|  | Mood & anxiety disorders ➞ Other solid cancer |
|  | Osteoarthritis ➞ Other solid cancer |
| Cluster 24: Heart disease | |
| Cluster prevalence (per 100) | 0.001 |
| Average link weight in cluster | 3.406 |
| Cluster links | Asthma ➞ Heart disease |
|  | Diabetes ➞ Heart disease |
|  | Hypertension ➞ Heart disease |
|  | Mood & anxiety disorders ➞ Heart disease |
|  | Osteoarthritis ➞ Heart disease |
|  | Osteoporosis ➞ Heart disease |
| Cluster 25: Multiple sclerosis | |
| Cluster prevalence (per 100) | 0.001 |
| Average link weight in cluster | 3.041 |
| Cluster links | Multiple sclerosis ➞ COPD |
|  | Multiple sclerosis ➞ Heart disease |
|  | Multiple sclerosis ➞ Osteoporosis |
| Cluster 26: Breast cancer | |
| Cluster prevalence (per 100) | 0.001 |
| Average link weight in cluster | 3.072 |
| Cluster links | Asthma ➞ Breast cancer |
|  | Diabetes ➞ Breast cancer |
|  | Hypertension ➞ Breast cancer |
|  | Mood & anxiety disorders ➞ Breast cancer |
|  | Osteoarthritis ➞ Breast cancer |
| Cluster 27: Lung cancer | |
| Cluster prevalence (per 100) | 0.001 |
| Average link weight in cluster | 3.121 |
| Cluster links | Asthma ➞ Lung cancer |
|  | Diabetes ➞ Lung cancer |
|  | Hypertension ➞ Lung cancer |
|  | Mood & anxiety disorders ➞ Lung cancer |
|  | Osteoarthritis ➞ Lung cancer |
| **Males** | |
| Number of clusters: 13 | |
| Cluster 1: Osteoarthritis | |
| Cluster prevalence (per 100) | 0.654 |
| Average link weight in cluster | 3.71 |
| Cluster links | Hypertension ➞ Osteoarthritis |
|  | Mood & anxiety disorders ➞ Osteoarthritis |
| Cluster 2: Diabetes + Hypertension + Mood & anxiety disorders | |
| Cluster prevalence (per 100) | 0.585 |
| Average link weight in cluster | 3.764 |
| Cluster links | Hypertension ➞ Diabetes |
|  | Mood & anxiety disorders ➞ Diabetes |
|  | Mood & anxiety disorders ➞ Hypertension |
| Cluster 3: Gout | |
| Cluster prevalence (per 100) | 0.041 |
| Average link weight in cluster | 3.787 |
| Cluster links | Gout ➞ Diabetes |
|  | Hypertension ➞ Gout |
|  | Mood & anxiety disorders ➞ Gout |
| Cluster 4: Haematological cancer | |
| Cluster prevalence (per 100) | 0.03 |
| Average link weight in cluster | 3.333 |
| Cluster links | Hypertension ➞ Haematological cancer |
|  | Mood & anxiety disorders ➞ Haematological cancer |
| Cluster 5: Alzheimer's & dementia | |
| Cluster prevalence (per 100) | 0.016 |
| Average link weight in cluster | 6.424 |
| Cluster links | COPD ➞ Alzheimer's & dementia |
|  | Heart failure ➞ Alzheimer's & dementia |
| Cluster 6: COPD | |
| Cluster prevalence (per 100) | 0.012 |
| Average link weight in cluster | 8.772 |
| Cluster links | COPD ➞ Chronic kidney disease |
|  | COPD ➞ Lung cancer |
| Cluster 7: Melanoma cancer | |
| Cluster prevalence (per 100) | 0.011 |
| Average link weight in cluster | 3.501 |
| Cluster links | Hypertension ➞ Melanoma cancer |
|  | Mood & anxiety disorders ➞ Melanoma cancer |
| Cluster 8: Heart disease | |
| Cluster prevalence (per 100) | 0.007 |
| Average link weight in cluster | 3.564 |
| Cluster links | Diabetes ➞ Heart disease |
|  | Gout ➞ Heart disease |
|  | Hypertension ➞ Heart disease |
|  | Mood & anxiety disorders ➞ Heart disease |
|  | Osteoarthritis ➞ Heart disease |
| Cluster 9: Heart failure | |
| Cluster prevalence (per 100) | 0.007 |
| Average link weight in cluster | 6.795 |
| Cluster links | Heart failure ➞ Chronic kidney disease |
|  | Heart failure ➞ Lung cancer |
| Cluster 10: Chronic kidney disease | |
| Cluster prevalence (per 100) | 0.002 |
| Average link weight in cluster | 8.351 |
| Cluster links | Colorectal cancer ➞ Chronic kidney disease |
|  | Other solid cancer ➞ Chronic kidney disease |
| Cluster 11: Rheumatoid arthritis | |
| Cluster prevalence (per 100) | 0.001 |
| Average link weight in cluster | 3.816 |
| Cluster links | Rheumatoid arthritis ➞ Chronic kidney disease |
|  | Rheumatoid arthritis ➞ Lung cancer |
| Cluster 12: Asthma | |
| Cluster prevalence (per 100) | 0.001 |
| Average link weight in cluster | 2.636 |
| Cluster links | Asthma ➞ Colorectal cancer |
|  | Asthma ➞ Other solid cancer |
| Cluster 13: Schizophrenia | |
| Cluster prevalence (per 100) | 0.001 |
| Average link weight in cluster | 3.695 |
| Cluster links | Mood & anxiety disorders ➞ Schizophrenia |
|  | Schizophrenia ➞ Diabetes |
|  | Schizophrenia ➞ Heart disease |
|  | Schizophrenia ➞ Hypertension |
|  | Schizophrenia ➞ Osteoarthritis |
| **70+** | |
| **Females** | |
| Number of clusters: 35 | |
| Cluster 1: Hypertension + Mood & anxiety disorders + Osteoporosis | |
| Cluster prevalence (per 100) | 0.767 |
| Average link weight in cluster | 5.878 |
| Cluster links | Hypertension ➞ Mood & anxiety disorders |
|  | Hypertension ➞ Osteoporosis |
|  | Osteoporosis ➞ Mood & anxiety disorders |
| Cluster 2: Chronic kidney disease | |
| Cluster prevalence (per 100) | 0.551 |
| Average link weight in cluster | 6.691 |
| Cluster links | Asthma ➞ Chronic kidney disease |
|  | Diabetes ➞ Chronic kidney disease |
| Cluster 3: Heart disease | |
| Cluster prevalence (per 100) | 0.422 |
| Average link weight in cluster | 5.616 |
| Cluster links | Hypertension ➞ Heart disease |
|  | Mood & anxiety disorders ➞ Heart disease |
|  | Osteoporosis ➞ Heart disease |
| Cluster 4: Heart failure | |
| Cluster prevalence (per 100) | 0.419 |
| Average link weight in cluster | 6.122 |
| Cluster links | Asthma ➞ Heart failure |
|  | Diabetes ➞ Heart failure |
| Cluster 5: Heart disease | |
| Cluster prevalence (per 100) | 0.366 |
| Average link weight in cluster | 5.886 |
| Cluster links | Asthma ➞ Heart disease |
|  | Diabetes ➞ Heart disease |
| Cluster 6: Diabetes | |
| Cluster prevalence (per 100) | 0.28 |
| Average link weight in cluster | 5.39 |
| Cluster links | Hypertension ➞ Diabetes |
|  | Mood & anxiety disorders ➞ Diabetes |
|  | Osteoporosis ➞ Diabetes |
| Cluster 7: Alzheimer's & dementia | |
| Cluster prevalence (per 100) | 0.215 |
| Average link weight in cluster | 6.349 |
| Cluster links | Hypertension ➞ Alzheimer's & dementia |
|  | Mood & anxiety disorders ➞ Alzheimer's & dementia |
|  | Osteoarthritis ➞ Alzheimer's & dementia |
|  | Osteoporosis ➞ Alzheimer's & dementia |
| Cluster 8: Asthma | |
| Cluster prevalence (per 100) | 0.179 |
| Average link weight in cluster | 5.439 |
| Cluster links | Hypertension ➞ Asthma |
|  | Mood & anxiety disorders ➞ Asthma |
|  | Osteoporosis ➞ Asthma |
| Cluster 9: Gout | |
| Cluster prevalence (per 100) | 0.141 |
| Average link weight in cluster | 6.786 |
| Cluster links | Asthma ➞ Gout |
|  | Diabetes ➞ Gout |
| Cluster 10: Chronic kidney disease + Heart failure + Other solid cancer | |
| Cluster prevalence (per 100) | 0.106 |
| Average link weight in cluster | 7.407 |
| Cluster links | Chronic kidney disease ➞ Other solid cancer |
|  | Heart failure ➞ Chronic kidney disease |
|  | Heart failure ➞ Other solid cancer |
| Cluster 11: Stroke | |
| Cluster prevalence (per 100) | 0.05 |
| Average link weight in cluster | 5.644 |
| Cluster links | Hypertension ➞ Stroke |
|  | Mood & anxiety disorders ➞ Stroke |
|  | Osteoarthritis ➞ Stroke |
|  | Osteoporosis ➞ Stroke |
| Cluster 12: Alzheimer's & dementia | |
| Cluster prevalence (per 100) | 0.046 |
| Average link weight in cluster | 5.941 |
| Cluster links | COPD ➞ Alzheimer's & dementia |
|  | Gout ➞ Alzheimer's & dementia |
|  | Heart disease ➞ Alzheimer's & dementia |
| Cluster 13: Breast cancer | |
| Cluster prevalence (per 100) | 0.045 |
| Average link weight in cluster | 6.078 |
| Cluster links | Asthma ➞ Breast cancer |
|  | Diabetes ➞ Breast cancer |
| Cluster 14: COPD | |
| Cluster prevalence (per 100) | 0.044 |
| Average link weight in cluster | 6.794 |
| Cluster links | COPD ➞ Chronic kidney disease |
|  | COPD ➞ Heart failure |
|  | COPD ➞ Other solid cancer |
| Cluster 15: Colorectal cancer | |
| Cluster prevalence (per 100) | 0.036 |
| Average link weight in cluster | 5.772 |
| Cluster links | Hypertension ➞ Colorectal cancer |
|  | Mood & anxiety disorders ➞ Colorectal cancer |
|  | Osteoporosis ➞ Colorectal cancer |
| Cluster 16: Colorectal cancer | |
| Cluster prevalence (per 100) | 0.033 |
| Average link weight in cluster | 5.944 |
| Cluster links | Asthma ➞ Colorectal cancer |
|  | Diabetes ➞ Colorectal cancer |
| Cluster 17: Gout | |
| Cluster prevalence (per 100) | 0.027 |
| Average link weight in cluster | 5.279 |
| Cluster links | Heart disease ➞ Gout |
|  | Hypertension ➞ Gout |
|  | Mood & anxiety disorders ➞ Gout |
|  | Osteoporosis ➞ Gout |
| Cluster 18: Haematological cancer | |
| Cluster prevalence (per 100) | 0.025 |
| Average link weight in cluster | 5.81 |
| Cluster links | Hypertension ➞ Haematological cancer |
|  | Mood & anxiety disorders ➞ Haematological cancer |
|  | Osteoporosis ➞ Haematological cancer |
| Cluster 19: Lung cancer | |
| Cluster prevalence (per 100) | 0.024 |
| Average link weight in cluster | 5.865 |
| Cluster links | Hypertension ➞ Lung cancer |
|  | Mood & anxiety disorders ➞ Lung cancer |
|  | Osteoarthritis ➞ Lung cancer |
|  | Osteoporosis ➞ Lung cancer |
| Cluster 20: Stroke | |
| Cluster prevalence (per 100) | 0.021 |
| Average link weight in cluster | 6.772 |
| Cluster links | COPD ➞ Stroke |
|  | Gout ➞ Stroke |
|  | Heart disease ➞ Stroke |
| Cluster 21: Haematological cancer | |
| Cluster prevalence (per 100) | 0.019 |
| Average link weight in cluster | 5.614 |
| Cluster links | Asthma ➞ Haematological cancer |
|  | Diabetes ➞ Haematological cancer |
| Cluster 22: Stroke | |
| Cluster prevalence (per 100) | 0.018 |
| Average link weight in cluster | 7.441 |
| Cluster links | Heart failure ➞ Stroke |
|  | Stroke ➞ Other solid cancer |
| Cluster 23: Lung cancer | |
| Cluster prevalence (per 100) | 0.014 |
| Average link weight in cluster | 9.421 |
| Cluster links | COPD ➞ Lung cancer |
|  | Gout ➞ Lung cancer |
|  | Heart disease ➞ Lung cancer |
| Cluster 24: Alzheimer's & dementia | |
| Cluster prevalence (per 100) | 0.01 |
| Average link weight in cluster | 5.025 |
| Cluster links | Asthma ➞ Alzheimer's & dementia |
|  | Diabetes ➞ Alzheimer's & dementia |
|  | Rheumatoid arthritis ➞ Alzheimer's & dementia |
| Cluster 25: Schizophrenia | |
| Cluster prevalence (per 100) | 0.007 |
| Average link weight in cluster | 5.73 |
| Cluster links | Hypertension ➞ Schizophrenia |
|  | Mood & anxiety disorders ➞ Schizophrenia |
|  | Osteoarthritis ➞ Schizophrenia |
|  | Osteoporosis ➞ Schizophrenia |
| Cluster 26: Stroke | |
| Cluster prevalence (per 100) | 0.005 |
| Average link weight in cluster | 5.457 |
| Cluster links | Asthma ➞ Stroke |
|  | Diabetes ➞ Stroke |
|  | Rheumatoid arthritis ➞ Stroke |
| Cluster 27: Heart failure | |
| Cluster prevalence (per 100) | 0.005 |
| Average link weight in cluster | 6.473 |
| Cluster links | Gout ➞ Heart failure |
|  | Heart disease ➞ Heart failure |
|  | Hypertension ➞ Heart failure |
|  | Mood & anxiety disorders ➞ Heart failure |
|  | Osteoarthritis ➞ Heart failure |
|  | Osteoporosis ➞ Heart failure |
| Cluster 28: Chronic kidney disease | |
| Cluster prevalence (per 100) | 0.005 |
| Average link weight in cluster | 6.445 |
| Cluster links | Gout ➞ Chronic kidney disease |
|  | Heart disease ➞ Chronic kidney disease |
|  | Hypertension ➞ Chronic kidney disease |
|  | Mood & anxiety disorders ➞ Chronic kidney disease |
|  | Osteoarthritis ➞ Chronic kidney disease |
|  | Osteoporosis ➞ Chronic kidney disease |
| Cluster 29: Other solid cancer | |
| Cluster prevalence (per 100) | 0.003 |
| Average link weight in cluster | 5.948 |
| Cluster links | Asthma ➞ Other solid cancer |
|  | Diabetes ➞ Other solid cancer |
|  | Rheumatoid arthritis ➞ Other solid cancer |
| Cluster 30: Osteoarthritis | |
| Cluster prevalence (per 100) | 0.003 |
| Average link weight in cluster | 6.032 |
| Cluster links | Osteoarthritis ➞ Colorectal cancer |
|  | Osteoarthritis ➞ Haematological cancer |
| Cluster 31: Lung cancer | |
| Cluster prevalence (per 100) | 0.003 |
| Average link weight in cluster | 7.217 |
| Cluster links | Chronic kidney disease ➞ Lung cancer |
|  | Heart failure ➞ Lung cancer |
|  | Other solid cancer ➞ Lung cancer |
| Cluster 32: Lung cancer | |
| Cluster prevalence (per 100) | 0.002 |
| Average link weight in cluster | 7.001 |
| Cluster links | Asthma ➞ Lung cancer |
|  | Diabetes ➞ Lung cancer |
|  | Rheumatoid arthritis ➞ Lung cancer |
| Cluster 33: Osteoarthritis | |
| Cluster prevalence (per 100) | 0.002 |
| Average link weight in cluster | 5.914 |
| Cluster links | Hypertension ➞ Osteoarthritis |
|  | Mood & anxiety disorders ➞ Osteoarthritis |
|  | Osteoarthritis ➞ Gout |
|  | Osteoarthritis ➞ Heart disease |
|  | Osteoporosis ➞ Osteoarthritis |
| Cluster 34: Chronic kidney disease | |
| Cluster prevalence (per 100) | 0.001 |
| Average link weight in cluster | 7.252 |
| Cluster links | Chronic kidney disease ➞ Colorectal cancer |
|  | Chronic kidney disease ➞ Haematological cancer |
| Cluster 35: Heart disease | |
| Cluster prevalence (per 100) | 0.001 |
| Average link weight in cluster | 5.839 |
| Cluster links | Heart disease ➞ Colorectal cancer |
|  | Heart disease ➞ Haematological cancer |
| **Males** | |
| Number of clusters: 11 | |
| Cluster 1: Diabetes + Gout + Osteoarthritis | |
| Cluster prevalence (per 100) | 0.235 |
| Average link weight in cluster | 5.795 |
| Cluster links | Diabetes ➞ Gout |
|  | Diabetes ➞ Osteoarthritis |
|  | Osteoarthritis ➞ Gout |
| Cluster 2: Hypertension | |
| Cluster prevalence (per 100) | 0.109 |
| Average link weight in cluster | 6.333 |
| Cluster links | Hypertension ➞ Diabetes |
|  | Hypertension ➞ Gout |
|  | Hypertension ➞ Heart disease |
|  | Hypertension ➞ Osteoarthritis |
| Cluster 3: Heart disease | |
| Cluster prevalence (per 100) | 0.05 |
| Average link weight in cluster | 5.632 |
| Cluster links | Diabetes ➞ Heart disease |
|  | Heart disease ➞ Gout |
|  | Osteoarthritis ➞ Heart disease |
| Cluster 4: Schizophrenia | |
| Cluster prevalence (per 100) | 0.03 |
| Average link weight in cluster | 7.181 |
| Cluster links | Heart disease ➞ Schizophrenia |
|  | Hypertension ➞ Schizophrenia |
|  | Mood & anxiety disorders ➞ Schizophrenia |
| Cluster 5: Alzheimer's & dementia | |
| Cluster prevalence (per 100) | 0.016 |
| Average link weight in cluster | 8.167 |
| Cluster links | Prostate cancer ➞ Alzheimer's & dementia |
|  | Stroke ➞ Alzheimer's & dementia |
| Cluster 6: Rheumatoid arthritis | |
| Cluster prevalence (per 100) | 0.01 |
| Average link weight in cluster | 7.028 |
| Cluster links | Diabetes ➞ Rheumatoid arthritis |
|  | Gout ➞ Rheumatoid arthritis |
|  | Hypertension ➞ Rheumatoid arthritis |
|  | Osteoarthritis ➞ Rheumatoid arthritis |
| Cluster 7: Osteoporosis | |
| Cluster prevalence (per 100) | 0.006 |
| Average link weight in cluster | 5.252 |
| Cluster links | Diabetes ➞ Osteoporosis |
|  | Gout ➞ Osteoporosis |
|  | Heart disease ➞ Osteoporosis |
|  | Hypertension ➞ Osteoporosis |
|  | Osteoarthritis ➞ Osteoporosis |
| Cluster 8: Mood & anxiety disorders | |
| Cluster prevalence (per 100) | 0.006 |
| Average link weight in cluster | 5.494 |
| Cluster links | Hypertension ➞ Mood & anxiety disorders |
|  | Mood & anxiety disorders ➞ Asthma |
|  | Mood & anxiety disorders ➞ Gout |
|  | Mood & anxiety disorders ➞ Osteoarthritis |
| Cluster 9: Asthma | |
| Cluster prevalence (per 100) | 0.004 |
| Average link weight in cluster | 7.021 |
| Cluster links | Asthma ➞ COPD |
|  | Asthma ➞ Gout |
|  | Asthma ➞ Osteoporosis |
|  | Hypertension ➞ Asthma |
| Cluster 10: Multiple sclerosis | |
| Cluster prevalence (per 100) | 0.003 |
| Average link weight in cluster | 6.011 |
| Cluster links | Multiple sclerosis ➞ Alzheimer's & dementia |
|  | Multiple sclerosis ➞ Chronic kidney disease |
| Cluster 11: Heart failure | |
| Cluster prevalence (per 100) | 0.002 |
| Average link weight in cluster | 6.273 |
| Cluster links | Diabetes ➞ Heart failure |
|  | Gout ➞ Heart failure |
|  | Heart disease ➞ Heart failure |
|  | Hypertension ➞ Heart failure |
|  | Osteoarthritis ➞ Heart failure |
|  | Rheumatoid arthritis ➞ Heart failure |
| Summary information for disease clusters identified from age- and sex-stratified lift networks. Clusters are named for the most frequently appearing disease(s) in cluster links. | |
